# Advanced Clinical-Based Technologies for Monitoring Physical Function in Breast Cancer Survivors: Scoping Review

**DOI:** 10.2196/77894

**Published:** 2026-01-14

**Authors:** Mahtab Azhdar, Adalberto Loyola-Sanchez, Amber Wardrop, Martin Ferguson-Pell

**Affiliations:** 1 Rehabilitation Sciences Rehabilitation Medicine University of Alberta Edmonton, AB Canada; 2 Division of Physical Medicine and Rehabilitation, Department of Medicine Faculty of Medicine and Dentistry University of Alberta Edmonton, AB Canada; 3 Department of Physical Therapy Faculty of Rehabilitation Medicine University of Alberta Edmonton, AB Canada

**Keywords:** breast cancer, technology, functional assessment, markerless motion capture, isokinetic dynamometry

## Abstract

**Background:**

People surviving breast cancer often face long-term impairments in physical function, significantly impacting their quality of life. In recent years, a variety of technologies have been developed to monitor and assess these functions; however, there is no consolidated synthesis linking specific technologies to targeted functional domains and real-world clinical contexts, limiting comparability and translation into practice.

**Objective:**

This scoping review aimed to systematically explore and map the use of advanced clinic-based technologies for assessing and monitoring key physical functions, such as balance, muscle strength, and range of motion, among individuals surviving breast cancer. The purpose of this review was not only to identify which technologies have been applied but also to clarify how they are being used, the clinical settings, target physical functions, assessment protocols, and types of outcomes measured. It further summarized the current patterns of use to inform and enhance clinical assessment practices.

**Methods:**

A comprehensive literature search was conducted across MEDLINE, Scopus, CINAHL, and Web of Science databases, with no publication date restrictions. Eligible studies included adults with breast cancer assessed using advanced clinic-based technologies to monitor physical function. Screening and selection followed PRISMA (Preferred Reporting Items for Systematic Reviews and Meta-Analyses) guidelines. The data extraction captured study characteristics, participant demographics, technologies applied, and related outcomes. The extracted data were organized in Covidence and synthesized descriptively to map the types of technologies, assessed functional domains, and application settings across studies.

**Results:**

Across the 17 included studies, the participants (N=719; age range between 30 and 75 years) were predominantly female and largely drawn from stage 0 to III breast cancer cohorts; 1 (5.9%) study reported a single male participant, and 2 (11.8%) studies did not specify participant sex. Among the 17 included studies, 11 (64.7%) were published from 2017 onward. Technologies spanned balance platforms (force plates, Technobody-PK 200 WL, Sensory Organization Test; 5/17, 29.4%), isokinetic dynamometry (Biodex systems; 4/17, 23.5%), and range of motion assessment via motion capture (3/17, 17.6%) or digital inclinometers (5/17, 29.4%). Sample sizes per study ranged from 20 to 100 participants (median 43), and follow-up durations varied from 1 session to 6 months.

**Conclusions:**

Advanced clinic-based technologies for assessing balance, muscle strength, and range of motion in breast cancer survivors were identified across the literature, including balance platforms, isokinetic dynamometry, digital inclinometers, and markerless motion capture systems. Considerable heterogeneity in devices, outcome reporting, and study designs limited direct comparison across studies and prevented definitive conclusions about the superiority or clinical readiness of any single technology.

## Introduction

Breast cancer remains the most prevalent malignancy among women worldwide, with increasing survival rates due to advances in early detection and treatment [1]. Although breast cancer mortality has declined in recent decades, as survival rates improve, attention has shifted from survival alone to long-term recovery, quality of life, and function, with rehabilitation emerging as a central component of survivorship care [2-4]. Physical function, the ability to perform physical tasks that enable daily activities and participation, is an important factor in cancer survivorship and rehabilitation and is framed within the International Classification of Functioning, Disability, and Health (ICF) [5]. Impairments in physical function after breast cancer treatment are commonly reported and include deficits in balance, muscle strength, and range of motion (ROM) [6-8]. Women undergoing chemotherapy may experience up to a 25% loss in strength and joint dysfunction [8]. Moreover, individuals surviving cancer have highlighted difficulties with balance and walking as the most common functional issues, with prevalence rates of 19% and 24%, respectively [9]. These functional limitations have been associated with poorer health-related quality of life and reduced mobility in survivorship cohorts [10,11]. At the same time, impairments in gait and balance control have been documented as potential contributors to fall risk and reduced independence in everyday life among breast cancer survivors [6]. According to the literature on cancer survivorship, over half of individuals who have undergone cancer treatments encounter physical function impairments [12].

The American Physical Therapy Association (APTA) Oncology Evaluation Database to Guide Effectiveness (EDGE) Task Force has provided evidence-based recommendations for standardized outcome measures in oncology rehabilitation. For balance assessment, the Task Force strongly supports the use of low-cost, performance-based tools such as the Fullerton Advanced Balance Scale, gait speed, Timed Up and Go, Five Times Sit-to-Stand, and the Balance Evaluation Systems Test, all rated as reliable and clinically feasible for cancer survivors [13]. In contrast, computerized balance systems such as force plates and the Sensory Organization Test (SOT) have been less recommended due to limited clinical utility and high cost, despite growing evidence supporting their sensitivity in detecting subtle postural sway and vestibular deficits in individuals with cancer and chemotherapy-induced peripheral neuropathy [14]. For shoulder ROM, the Task Force rated passive goniometry (score 4) as a recommended tool, while for muscle strength, handheld dynamometers (HHDs) (score 3) and manual muscle testing (score 2B) were endorsed as appropriate clinical measures [15]. Although these conventional methods remain the clinical standard, their limited sensitivity and responsiveness underscore the need for more objective, automated technologies capable of quantifying subtle changes in function [16,17].

Furthermore, a variety of technologies such as wearable sensors (accelerometers/pedometers), fitness trackers, smartphone apps, and advanced motion-capture systems are increasingly used to quantify physical function in breast cancer survivorship [18-22], providing objective, high-resolution data [21,22]. However, most reported applications remain in research or specialized settings with limited protocol and outcome standardization [19,22].

Accordingly, this review aims to map and characterize the use of such advanced, clinically based technologies in assessing key physical functions, including balance, muscle strength, and ROM, among individuals surviving breast cancer. For the purposes of this review, “advanced” is defined as instruments that provide automated, objective outputs beyond unaided observation or analogue readouts. “Clinic-based” denotes systems that can be operated in clinical rooms or clinically configured spaces by routine clinical staff with minimal specialist engineering support, for monitoring and assessing physical functions. These terms were selected based on their relevance within the ICF framework and their frequent association with impairment among breast cancer survivors [6,23,24]. By examining how these technologies are currently integrated into clinical practice, this review seeks to identify gaps in the existing literature and highlight areas where further research is needed.

## Methods

### Search Strategy

A comprehensive literature search was conducted across the MEDLINE, Scopus, CINAHL, and Web of Science databases. The initial search strategy was developed in collaboration with an experienced academic health sciences librarian (Liz Dennett) to ensure methodological rigor and comprehensive coverage. The selection of the search terms was informed by preliminary scoping searches and key indexing terms from prior reviews in oncology rehabilitation and motion analysis. The MEDLINE strategy served as the base and was adapted for syntax variations across databases. The search was conducted in multiple stages, starting with the initial search on February 2, 2024, followed by updates on June 24, 2024. Each search was repeated across the 4 databases to ensure comprehensive coverage of the relevant literature. The final search included combinations of controlled vocabulary, such as MeSH (Medical Subject Headings) terms and free-text terms related to breast cancer, physical function, and measurement technologies. The complete search strategy and related keywords are provided in Multimedia Appendix 1. In addition to peer-reviewed databases, gray-literature searches were limited to PhD dissertations. All identified articles were imported into Covidence software (Covidence Ltd) for screening. Duplicate records were automatically identified and removed by Covidence’s built-in algorithm, followed by manual verification by the reviewers to ensure accuracy.

### Inclusion and Exclusion Criteria

Studies were included if they were published in English, had full-text availability online, involved adults diagnosed with breast cancer, and utilized technologies to objectively measure one or more physical functions (balance, strength, and ROM) as primary or secondary outcomes. No publication date restrictions were applied. All study designs were considered for inclusion, excluding nonoriginal articles such as study protocols, reviews, conference abstracts, books, or editorials. Additionally, studies were excluded if they involved technologies that did not meet the criteria for being both advanced and clinically based. These technologies are intended to directly assess and monitor physical functions in patients, facilitating clinical decision-making and supporting rehabilitation processes. Technologies like Vicon, for instance, were excluded if they were not designed for clinical use. To be effective in clinical settings, technologies must be easily integrable into workflows without requiring complex setups or specialized technical expertise.

### Screening and Selection Process

An initial search strategy was developed in collaboration with the academic librarian to capture studies using any technology to assess physical function in adults with breast cancer. During the title and abstract screening process, it became evident that this comprehensive approach would yield several hundred eligible studies, predominantly due to the extensive literature on accelerometers and pedometers, as well as the frequent application of laboratory-based marker-based motion-capture systems. To preserve methodological rigor and avoid redundancy with existing systematic reviews and meta-analyses focused on accelerometer and pedometer studies, while ensuring a clinically actionable synthesis, an additional consultation with the librarian was conducted to refine the eligibility criteria prior to initiating full-text review and data extraction. The inclusion criteria were subsequently refined to prioritize advanced, clinically deployable technologies for point-of-care assessment of 3 core domains of physical functions. As a result, articles that used traditional technologies, such as dynamometers or goniometers, which do not meet the established definition of advanced measurement technology for the purposes of this review; nonclinic–based accelerometers and pedometers; and some lab-based motion capture systems requiring reflective markers, multicamera stereophotogrammetry, calibration routines, and dedicated laboratory infrastructure, were excluded unless the authors explicitly described clinical deployment or adaptation for real-world clinical settings. The study selection process was conducted in several stages following established scoping review methodology. Initially, two reviewers (authors MA and AW) independently screened titles and abstracts of all retrieved references against the eligibility criteria. Discrepancies between the two reviewers at this stage were flagged within Covidence for subsequent resolution. Studies deemed potentially eligible by either reviewer advanced to full-text screening, which was similarly conducted independently by the same two reviewers (MA and AW). Following both screening phases, conflicts were systematically resolved through a structured consensus process. For any study where the two primary reviewers disagreed on inclusion or exclusion, the full text and relevant eligibility criteria were reviewed collaboratively by all three reviewers (MA, AW, and MFP) in a consensus meeting. During these discussions, each reviewer presented their rationale, and discrepancies were resolved through deliberation until unanimous agreement was reached. If consensus could not be achieved through discussion, the third reviewer (MFP) served as the final arbiter. All final inclusion decisions and data extraction were completed through this consensus process, with regular meetings held among the three reviewers to ensure consistency in the interpretation of eligibility criteria and data extraction procedures throughout the review.

### Data Extraction

Data extraction was collaboratively performed by the three reviewers (MA, AW, and MFP). For each included study, details regarding the study title, year of publication, country, aim, and design were recorded. Participant characteristics, including demographic information, inclusion and exclusion criteria, and recruitment methods and settings, were extracted. Information on sample size, cancer treatment details, and the instruments employed, including technological characteristics, was collected. We also recorded methodological features relevant to implementation and key findings reported by the original authors in relation to the use or outcomes of the measurement technology. The extracted data were organized and managed in Covidence for transparency and synthesis.

### Data Synthesis

The extracted data from relevant studies were synthesized narratively and summarized in tabular format to provide a comprehensive overview of advanced clinic-based technologies used to monitor and assess functional outcomes in individuals surviving breast cancer. Studies were first categorized by the component of physical function assessed (balance, strength, and ROM) and then organized according to the specific measurement technology employed. For each technology identified, the corresponding data analysis methods used in the original studies were extracted and documented. This synthesis sought to deliver an analysis of the current knowledge and methodologies used to assess the included physical functions. The findings were analyzed to highlight common methodologies, instruments, and outcomes reported in the literature. This process defines how functional outcomes are commonly evaluated in individuals with breast cancer using advanced and clinic-based technologies and identifies potential gaps or inconsistencies in current practices, including variations in measurement protocols, differences in data normalization methods, and the range of technologies applied to assess similar functional outcomes.

## Results

### Study Identification

**Figure 1:**
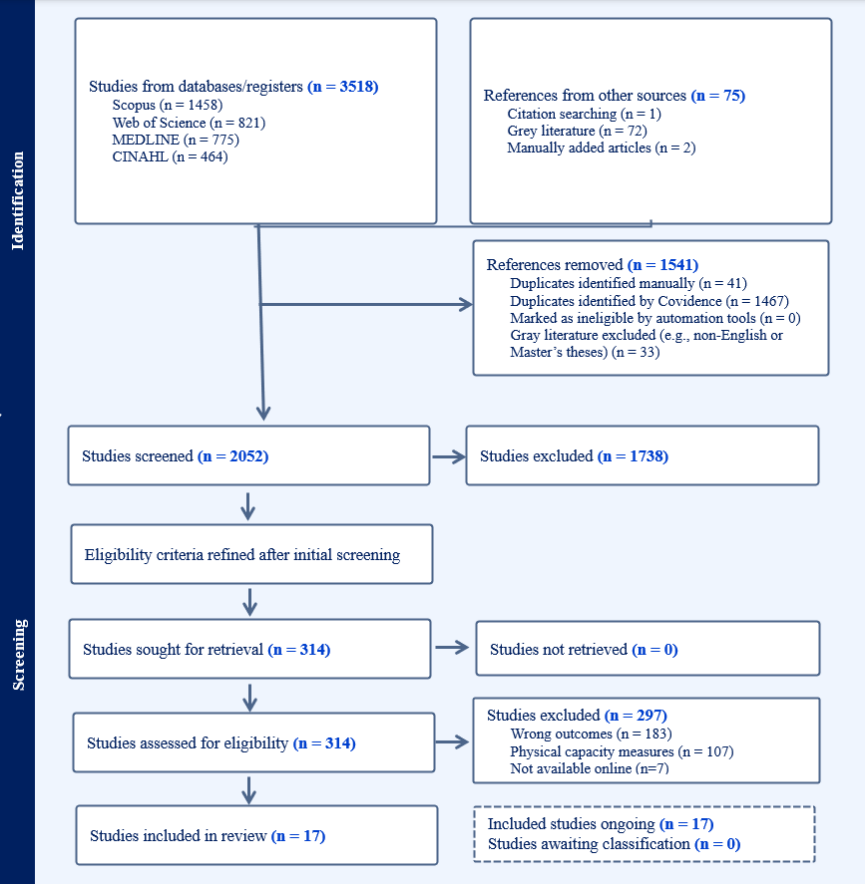
PRISMA (Preferred Reporting Items for Systematic Reviews and Meta-Analyses) 2020 flow diagram for study selection.

Initially, 3593 articles were retrieved from Medline, Scopus, Web of Science, CINAHL, and gray literature. After removing duplicates and nonrelevant gray literature (n=1508), a total of 2052 records were screened for eligibility. Following title and abstract screening, 314 articles were eligible for full-text review. Ultimately, 17 studies, each assigned a unique study ID (ranging from 001 to 017), were included for data extraction. These 17 studies, involving a total of 719 participants, were included in the review. The number of articles and methodologies utilized for reviewing, selecting, and verifying them at each stage of the process is presented in a PRISMA (Preferred Reporting Items for Systematic Reviews and Meta-Analyses) flow diagram (Figure 1) [25]. While gray literature, including PhD dissertations, was considered, none met the inclusion criteria. Furthermore, the specific characteristics of the studies included in this study are comprehensively detailed in Table 1, providing an in-depth overview of their methodologies, sample populations, and key findings. Additionally, no formal quality assessment of the studies was conducted as part of this scoping review.

**Table 1:** Characteristics of the included studies.

ID	Author (s)	Pub. Year	Title	Study setting and design	Sample size and participant characteristics	Physical function	Instruments	Key findings
001	(Bertoli et al) [26]	2023	Mat Pilates improves lower and upper body strength and flexibility in breast cancer survivors undergoing hormone therapy: a randomized controlled trial (HAPiMat study).	Hospital and randomized controlled trial	n=43; stage 0-III; ≥40 years; undergoing hormone therapy; sex not reported	Hip flexor and extensor muscle strength	Biodex Medical system 4 (Shirley)	Biodex assessments showed that the Pilates group significantly improved their lower body strength, with increases in isometric flexor-extensor peak torque and enhanced concentric and eccentric flexor peak torque and mechanical work.
002	(Rao and Pattanshetty) [27]	2022	Effect of myofascial release, stretching, and strengthening on upper torso posture, spinal curvatures, range of motion, strength, shoulder pain and disability, and quality of life in breast cancer survivors.	Hospital and pre‐post experimental study	n=22; female, 35-70 years; newly diagnosed; treated with surgery, radiation, chemotherapy, or combination	Shoulder and cervical range of motion	Cervical range of motion was evaluated using a digital inclinometer	Significant improvements were observed for cervical movements.
003	(Højvig et al) [28]	2022	Donor-site morbidity following breast reconstruction with a latissimus dorsi flap - A prospective study.	Hospital and prospective observational study	n=20; female, 32-70 years; undergoing delayed breast reconstruction with latissimus dorsi flap	Shoulder Strength	Biodex System4 Pro-dynamometer	The study revealed that breast reconstruction using the LD^a^ flap led to significant decreases in isometric shoulder strength for adduction and extension due to LD muscle removal, supporting its impact on shoulder girdle strength; however, isokinetic strength remained largely unchanged 12 weeks after surgery.
004	(Zabi̇t Özdemir and İyi̇gün) [29]	2022	Is there a difference in balance functions between breast cancer survivor women and healthy women?	Eastern Mediterranean University's Healthy Living Center and A prospective study	n=66; female, 35-70 years; 33 postmastectomy survivors vs 33 healthy controls; no chemo/radiation	Dynamic balance	Computer-based Dynamic Balance Platform (Technobody-PK 200 WL)	No differences were observed between the groups in subparameter dynamic balance measurements, computer-based dynamic balance platform assessments, or Y-Balance Test results.
005	(Wechsler et al) [30]	2022	Persistent cancer-related fatigue predicts static and dynamic balance in women with a history of breast cancer	A breast cancer center at a large urban hospital and cross-sectional study	n=43; female, 30-85 years; postchemotherapy ± radiation; stage I-III	Static and dynamic balance	Force plates (Bertec Corporation model 4060NC)	CRF^b^ independently and significantly impairs both static and dynamic balance individuals surviving cancer, leading to compensatory stabilization strategies and highlighting CRF's critical role in increased postural sway and fall risk even years after treatment.
006	(Artese et al) [31]	2021	Effect of functional impact training on body composition, bone mineral density, and strength in breast cancer survivors	Laboratory and randomized controlled trial	n=44; sedentary postmenopausal women, 52-68 years; stage 0-III; ≥3 months postchemotherapy/radiation.	Isokinetic concentric knee extension and flexion	Biodex Medical System 3 (Shirley)	Both groups demonstrated significant improvements over time in lower body strength, specifically in isokinetic knee extension and flexion at all tested speeds. However, after adjusting for baseline differences and time since diagnosis, there were no significant differences in posttraining knee strength measures between the two groups.
007	(Evans et al) [32]	2021	Examination of clinical and laboratory measures of static and dynamic balance in breast cancer survivors	In the Department of Rehabilitation Services at Alamance Regional Medical Center and a cross-sectional study	n=43 (20 breast cancer survivors + 23 controls); female, 40-70 years; stage 0-III; completed chemo/radiation ≤ 5 years	Static balance	SOT^c^ was conducted using the NeuroCom SMART Balance Master (Natus Medical, Pleasanton)	NeuroCom SOT equilibrium assessments revealed that individuals surviving breast cancer generally maintained similar postural stability to the healthy control group. However, they showed significantly impaired balance in Conditions 2 and 3, which rely on proprioceptive and vestibular systems for maintaining balance.
008	(Uhm et al) [33]	2020	Usefulness of Kinect sensor-based reachable workspace system for assessing upper extremity dysfunction in breast cancer patients	Konkuk University Medical Center and a cross-sectional study	n=20; age 46-62 years; unilateral breast cancer; sex not specified	Upper extremity active range of motion	The Kinect sensor-based reachable workspace analysis system	The reachable workspace was divided into four shoulder-centered quadrants: upper medial (Q1), lower medial (Q2), upper lateral (Q3), and lower lateral (Q4). The analysis revealed that the upper quadrants (1 and 3) on the affected side had significantly smaller reachable workspace areas compared to the unaffected side, while the lower quadrants showed no differences.
009	(Ribeiro et al) [34]	2019	Three-dimensional scapular kinematics, shoulder outcome measures and quality of life following treatment for breast cancer - a case control study	An outpatient breast cancer surgery (part of a hospital) and case control study	n=42; female, 40-60 years; 21 presurgery vs 21 controls	Shoulder range of motion	Shoulder motion measured by a digital inclinometer	The breast cancer surgery group exhibited reduced range of motion compared to healthy controls.
010	(Monfort et al) [35]	2017	Gait, balance, and patient-reported outcomes during taxane-based chemotherapy in early-stage breast cancer patients	An outpatient oncology clinic setting and longitudinal study	n=33; 32 female, 1 male; 36-59 years; Stage I-3	Static balance	Balance plate (Bertec Corp)	Cumulative exposure to taxane therapy was associated with notable declines in patients' balance, indicating a detrimental effect on their physical functionality.
011	(De Groef et al) [36]	2017	Effect of myofascial techniques for treatment of upper limb dysfunctions in breast cancer survivors: randomized controlled trial	Department of Physical Medicine and Rehabilitation of the University Hospitals Leuven and randomized controlled trial	n=48; female, 38-70 years; primary breast cancer; 23 intervention vs 25 control	Active shoulder range of motion	Inclinometer	The study found no significant differences between the group receiving myofascial therapy combined with physical therapy and the group receiving physical therapy alone in terms of shoulder range of motion.
012	(De Groef et al) [37]	2016	Arm lymphoedema and upper limb impairments in sentinel node-negative breast cancer patients: a one-year follow-up study	Multidisciplinary Breast Centre of University Hospitals Leuven and longitudinal study	n=100; female, 50-70 y	Shoulder range of motion	Gravity inclinometer	One year after the sentinel lymph node biopsy, 30% of patients experienced reduced shoulder range of motion.
013	(Moreira et al) [38]	2015	A Kinect-based system for upper-body function assessment in breast cancer patients	Not specified and an observational study	n=48; female; 24 with lymphedema; age not reported	Upper-body joint range of motion includes shoulder, elbow, and wrist flexion/extension and shoulder abduction/adduction	Kinect-based system	The study validated a Kinect-based system for assessing upper-body function, demonstrating high accuracy in classifying normal vs. impaired function. The system, which uses kinematic data for machine learning classification, shows potential for remote monitoring and early detection of functional impairments during rehabilitation.
014	(Gritsenko et al) [39]	2015	Feasibility of using low-cost motion capture for automated screening of shoulder motion limitation after breast cancer surgery	Academic cancer center oncology clinic and descriptive study	n=20; women, 51-69 years; stage 0-III	Active and passive shoulder range of motion	Motion capture by Kinect	The study found that the low-cost Kinect motion capture system effectively identified moderate to severe shoulder motion impairments in individuals surviving breast cancer, with strong correlations to goniometric measurements for active movements.
015	(Winters-Stone et al) [40]	2011	Identifying factors associated with falls in postmenopausal breast cancer survivors: a multi-disciplinary approach	Comprehensive cancer center and case-control plus prospective observation	n=59; female, 49-68 years; stage 0-III	Dynamic balance	The SOT, used in computerized dynamic posturography	Individuals surviving breast cancer had higher fall rates due to vestibular balance deficits from chemotherapy, particularly affecting dynamic balance, while static balance remained similar between fallers and non-fallers.
016	(Harrington et al) [41]	2011	Comparison of shoulder flexibility, strength, and function between breast cancer survivors and healthy participants	The Neuromuscular Research Laboratory and case-control study	n=48; female, 40-60 years; stage 0-III	Active and passive shoulder range of motion	Digital inclinometer	The study found that breast cancer survivors had reduced shoulder range of motion compared to healthy controls, particularly in flexion and external rotation.
017	(Waltman et al) [42]	2003	Testing an intervention for preventing osteoporosis in postmenopausal breast cancer survivors	In participants’ homes or at convenient sites and pilot intervention study	n=21; female, 40-65 years; stage I-II	Muscle strength of the knee, hip, and wrist (flexion and extension)	Biodex System 2 multijoint testing	The study found significant improvements in muscle strength for hip flexion, hip extension, and knee flexion over 12 months.

^a^LD: latissimus dorsi.

^b^CRF: cancer-related fatigue.

^c^SOT: Sensory Organization Test.

### Characteristics of the Included Studies

A review of 17 studies focusing on individuals surviving breast cancer revealed key insights into research locations, designs, timelines, participant stages, and objectives. The United States emerged as the most common location, hosting 7 (41.2%) of the included studies. The remaining research was conducted across a diverse set of countries, including Finland, Turkey, Portugal, Belgium, Brazil, South Korea, India, and Denmark. The review included 5 (29.4%) experimental studies, among which were 3 (17.6%) randomized controlled trials [26,31,36], 1 (5.9%) pretest-posttest experimental study [27], and 1 (5.9%) pilot intervention, [42] and 12 (70.6%) observational studies, including 4/17 (23.5%) cross-sectional studies [30,32,33,38], 3 (17.6%) case-control studies [34,40,41], 2 (11.8%) prospective observational studies [28,29], 2 (11.8%) longitudinal cohort studies [35,37], and 1 (5.9%) descriptive study [39]. The publication timeline indicated a growing interest in this area, with 11 (64.7%) studies published in 2017 or later, while the remaining 6 (35.3%) studies were published between 2003 and 2016 [37-42]. The studies aimed to assess different physical functions, with a particular emphasis on balance, strength, and flexibility related to breast cancer treatments. The objectives of the studies were varied, ranging from evaluating the effectiveness of specific interventions, such as mat Pilates [26] and myofascial techniques [36], to assessing the impact of cancer-related fatigue [30] on balance and postural control. Some studies also investigated the feasibility and usefulness of motion capture technologies for screening and assessing functional impairments [33,38,39].

Furthermore, the studies included in the review were conducted in multiple settings, predominantly within specialized medical facilities. Many studies took place in hospitals or hospital-affiliated centers. Isokinetic dynamometry using Biodex was reported mainly in hospital settings [26,28], with 1 (5.9%) study conducted in a laboratory environment [31]. Digital inclinometers were used across hospitals, rehabilitation services, and outpatient oncology clinics [34,36,37,41]. Kinect-based markerless motion capture (MMC) was implemented within oncology services spanning a medical center and an outpatient oncology clinic [33,38,39]. One (5.9%) study even extended its setting to participants’ homes or convenient community sites [42].

In terms of recruitment strategies, many studies enrolled participants through state and hospital cancer registries, clinician referrals, and ongoing treatment facilities within large urban hospitals. Researchers also leveraged community outreach by utilizing social media platforms, distributing flyers in regional cancer hospitals, and engaging with breast cancer support groups. Local media outlets, such as newspapers and radio announcements, were employed to broaden their reach. Some studies relied on word-of-mouth, emails, and telephone contacts.

The studies included in this review primarily focused on individuals surviving breast cancer, with participants aged between 30 and 75 years, and an average age range of 50 to 60 years. Among the 15 (88.2%) studies that reported participant sex, all included only female participants except for 1 (6.7%) study, which included a single male participant [35]. A total of 2 (11.8%) studies did not report any information about participant sex [26,33]. Nine (53%) of the included studies explicitly reported participants' cancer stage; all 9 enrolled individuals with stage 0 to III disease [26,30-32,35,39-42]. Among the 17 studies, 5 (29.4%) studies involved participants undergoing or having recently completed chemotherapy [27,30-32,41], and 1 (5.9%) study specifically enrolled women receiving hormone therapy [26]. Moreover, 1 (5.9%) study focused on surgical reconstruction (latissimus dorsi flap) [28]. Two (11.8%) studies included postmastectomy survivors compared to healthy controls [29,32], and 3 (17.6%) enrolled mixed-treatment cohorts [27,30,40]. In terms of sample sizes, these varied considerably throughout the studies, ranging from as few as 20 participants to as many as 100. People with stage IV cancer and cognitive impairments were generally excluded. Additional exclusion criteria, such as uncontrolled cardiovascular or musculoskeletal conditions, severe neuropathy, or inability to provide informed consent, were applied to ensure participant safety and maintain the validity of assessment results.

### Reported Instruments and Key Findings on Physical Function

This scoping review found that most studies assessed physical function using a variety of outcome measures, and ROM was reported in 8 (47.1%) of the included studies [27,33,34,36-39,41]. Balance outcomes were reported in 5 (29.4%) studies [29,30,32,35,40], and muscle strength outcomes were reported in 4 (23.5%) studies [26,28,31,42]. Of these, 3 (17.6%) studies employed motion capture technologies [33,38,39], Kinect-based systems to assess upper extremity kinematics and ROM in individuals surviving breast cancer. Inclinometers were used in 5 (29.4%) studies to further quantify shoulder movements [27,34,36,37,41]. Balance assessments were another key focus: 2 (11.8%) studies employed force plates to measure postural sway in both static and dynamic conditions [30,35], while the SOT was used in another 2 (11.8%) studies to assess balance under varying sensory inputs [32,40]. In addition, 1 (5.9%) prospective study evaluated dynamic balance using a computer-based dynamic balance platform (Technobody PK 200 WL) [29].

Moreover, strength assessments were conducted using different models of the Biodex System (2, 3, 4, and 4 Pro) to evaluate isometric and isokinetic strength in muscle groups, including the shoulder, hip, knee, and wrist [26,28,31,42]. Measurements included isometric strength normalized to body weight, as well as peak torque during isokinetic contractions at varying angles and velocities. Further details on the specific tools and data analysis methods used in each study are provided in Table 2.

**Table 2:** Overview of technologies and data analysis methods.

Category and technology used		Data analysis method	Author
**Strength**			
	Biodex System 2 multijoint testing	The highest peak torque measure, obtained during 4 repetitions, was recorded in Newton meters and used for data analysis.	Waltman et al [42]
	Biodex Medical System 3	The highest peak torque value from the 3 repetitions was recorded for each speed.	Artese et al [31]
	Biodex Medical Systems 4	Peak torque and mechanical work, normalized to body mass, were analyzed.	Bertoli et al [26]
	Biodex System 4 pro	The data were analyzed by averaging 3 trials of isometric and isokinetic strength (in Nm/kg).	Højvig et al [28]
	Digital inclinometer	Active and passive shoulder flexion/extension and external/internal rotation range of motion were measured with 3 trials averaged for data analysis.	Harrington et al [41]; Rao and Pattanshetty [27]; Ribeiro et al [34]
	Gravity inclinometer	The analysis assessed the prevalence of impaired shoulder range of motion, defined as an interlimb difference of 15 degrees or more between the affected and unaffected arms. Additionally, it evaluated the number of patients who experienced a decrease in shoulder ROM^a^ greater than 15 degrees from their baseline measurements, indicating a significant loss of mobility after treatment or surgery.	De Groef et al [36,37]
**Range of motion**			
	Kinect-based system	The study used a Kinect-based system to capture 3D motion data, extracting features like range of motion, hand height, elbow flexion, and movement acceleration. These were analyzed using machine learning algorithms to classify patients as having normal or impaired upper-body function.	Moreira et al [38]
	Automated motion analysis system using Microsoft Kinect	The Microsoft Kinect sensor was used to assess shoulder motion limitations. The technology tracked body landmarks and converted the captured data into joint angles using a custom algorithm.	Gritsenko et al [39]
	Microsoft Kinect Sensor	The Microsoft Kinect sensor captured participants' upper extremity motion trajectories during standardized seated arm movements. These data allowed for the reconstruction of each participant's reachable workspace envelope, divided into 4 quadrants relative to the shoulder joint. The reachable surface areas for each quadrant and the total workspace were calculated and then normalized by individual arm length to account for differences among participants.	Uhm et al [33]
	Force plate model 4060NC (Bertec Corp) and recorded through Motion Monitor Software (Innsport Training, Inc)	Balance was assessed using a force plate to measure postural sway in the medial-lateral and anterior-posterior planes under both static and dynamic conditions. For static balance evaluation, participants stood still for 30 seconds before and after moderate-intensity exercise. Dynamic balance was measured during the rising phase of an Instrumented Sit-to-Stand test, where participants quickly stood up from a seated position, and sway was recorded throughout this transition.	Wechsler et al [30]
**Balance**			
	Balance plate (Bertec Corp)	Data collected from the CoP^b^ included measurements of its location and displacement during standing trials, specifically focusing on the root mean squared excursion in the medial-lateral direction to evaluate postural stability and the risk of falling.	Monfort et al [35]
	Technobody-PK 200 WL, a computer-based dynamic balance platform	Participants performed the “Equilibrium Assessment” and “Sleight Assessment” tests on this device, which measured parameters such as anterior/posterior and medial/lateral sleight, balance assessments, number of targets reached, perimeter, and average pace. Each test was repeated 3 times, and the best score was recorded for analysis.	Zabi̇t Özdemi̇r and İyi̇gün [29]
	Computerized dynamic posturography with the SOT	Participants' sway responses were recorded under 6 sensory conditions that manipulate visual and somatosensory information.	Winters-Stone et al [40]
	NeuroCom SOT^c^	Equilibrium scores from the NeuroCom SOT under 6 conditions varying platform stability and visual input, with and without the serial sevens cognitive task, were analyzed to assess static balance and the impact of cognitive load.	Evans et al [32]

^a^ROM: range of motion.

^b^CoP: center of pressure.

^c^SOT: Sensory Organization Test.

## Discussion

### Principal Findings

This scoping review mapped 17 studies deploying advanced clinic-based technologies to assess physical function in breast cancer survivors. The review identified technologies used to assess three domains, namely ROM, balance, and muscle strength, and documented variation in instrument type. These technologies were applied in varied clinical environments, primarily hospital-based and outpatient oncology settings, to measure specific aspects of physical function through structured protocols.

The studies used advanced methods to determine how people maintain their balance. These detailed assessments consistently revealed information that simpler, one-time tests often overlook. Studies using advanced balance equipment in clinical settings measured different aspects of postural control and consistently revealed information that simpler, one-time tests often overlook. SOT protocols showed balance problems when proprioceptive and vestibular systems were challenged, even though performance was nearly normal under easier conditions. This suggests that balance deficits in breast cancer survivors depend on the situation rather than affecting all balance tasks equally [32,40]. This pattern supports the clinical value of multi-condition batteries that probe sensory reweighting, rather than relying solely on single-task screens. Force plates quantify quiet-stance stability by deriving center of pressure (CoP) signals and summarizing them with standard sway metrics, typically medial-lateral (ML) and anterior-posterior (AP) excursion (range or root mean square (RMS)), total path length, mean sway velocity, and planar sway area (often a 95% confidence ellipse). These metrics capture both the magnitude (eg, excursion, area) and temporal dynamics (eg, velocity) of postural control. Notably, key ML CoP metrics, including mean velocity, mean amplitude, and RMS displacement, have been linked to higher prospective fall risk [43]. In the included longitudinal study of taxane-based chemotherapy, Monfort et al [35] used a laboratory force plate to track changes in CoP behavior during treatment; cumulative taxane exposure was associated with deteriorations in balance control, reflected by increased sway and concurrent gait alterations (shorter step length, slower walking speed). Complementing this, Wechsler et al [30] showed that cancer-related fatigue independently predicted poorer postural stability on force-plate measurements. Fatigued individuals showed compensatory stabilization strategies and greater body sway in both standing still and moving conditions. Together, these findings suggest that when clinically relevant stressors are present (eg, neurotoxic chemotherapy and fatigue), force-plate CoP metrics could be sensitive to subtle balance changes.

For measuring ROM, the review describes a spectrum of technologies. At one end are digital inclinometers, which are widely accessible but depend on the operator. At the other end are automated MMC systems, which provide less biased 3D assessments and show promising evidence of clinical usefulness. The successful application of MMC for upper extremity ROM assessment in breast cancer survivors was demonstrated across 3 studies in diverse settings, including oncology clinics and outpatient centers. This suggests these technologies have overcome initial feasibility barriers and may be ready for broader clinical implementation [33,38,39]. The strong correlation between Kinect-derived reachable workspace metrics and QuickDASH, a validated and widely used tool for assessing upper extremity disability in breast cancer survivors [44], and the system’s ability to detect side-to-side differences in functionally relevant movement zones (overhead reaching) demonstrate practical utility for monitoring recovery [33]. However, there is significant variation in how different studies measure ROM. Studies differ in whether they assess active versus passive motion, which planes or tasks they evaluate, and whether they use absolute measurements versus side-to-side comparisons. This inconsistency makes it difficult to compare studies and develop clear, evidence-based clinical guidelines. Taken together, within the bounds of the included studies, MMC appears to have promising potential for assessing upper-extremity function in breast cancer survivors.

Muscle strength was quantified with isokinetic dynamometry in controlled clinical or laboratory settings [26,28,31,42]. Systems such as the Biodex (models 2-4/4 Pro) are seen as the top choice for testing dynamic muscle strength. They control precise, preset angular velocities and capture full torque-angle-velocity relationships together with work and power outputs. These multidimensional profiles can reveal deficits in force production and velocity-dependent behavior (eg, concentric versus eccentric weakness) that are not apparent from single-value manual grades, and their test-retest reliability in musculoskeletal [45,46]. The trade-off is practical; true isokinetic testing requires dedicated equipment, space, regular calibration, and staff skilled in positioning, stabilization, familiarization, and protocol standardization, all of which limit routine deployment outside well-resourced centers. Given these constraints, many rehabilitation services rely on portable HHDs for strength assessment. Professional guidance from the APTA Oncology EDGE Task Force recognizes HHDs as an appropriate option for cancer populations, provided that clinicians use standardized patient and tester positioning, consistent lever arms, and repeated trials to improve reliability [15].

This scoping review had some limitations. First, the inclusion of solely English-language studies raised the possibility of language bias and the exclusion of relevant work published in other languages. Additionally, the review focused solely on 3 physical functions, potentially overlooking other important aspects of physical function in individuals surviving breast cancer. While multiple databases were searched, some relevant studies may not have been captured if they were published in databases that were not included in the search strategy, which can limit the comprehensiveness of the review. Finally, the variability of the included studies in design, clinical setting, treatment phase, and outcome definitions (eg, active versus passive ROM, task/plane selection, and absolute versus interlimb metrics) precluded quantitative synthesis and limits cross-study comparability. Specifically, individuals with stage IV cancer were typically excluded from most of the included studies to focus on nonmetastatic cases.

### Comparison With Prior Work

A recent scoping review by Amarelo et al (2024) [47] examined the application of technological resources in cancer rehabilitation, identifying wearable devices, web-based platforms, mobile health (mHealth) apps, virtual reality, and exergaming as commonly employed tools. Their review emphasized the wide diversity of technologies and underscored the need for further research to assess their long-term effectiveness, cost-efficiency, and successful integration into clinical practice. In contrast, our scoping review narrows the focus to advanced clinic-based technologies specifically used to assess and monitor physical functions, balance, muscle strength, and ROM in individuals surviving breast cancer. By concentrating on this particular population and key physical functions, our review offers a detailed analysis of the clinical applicability, measurement properties, and limitations of these technologies. This focused approach addresses the gap identified by the previous work [47] regarding the need for more targeted research on the integration of technological tools into cancer rehabilitation practices. Moreover, our findings corroborate a previous review that recognizes force plates as effective tools for detecting subtle balance impairments [6] and Biodex systems as the gold standard for muscle strength assessment [48].

### Conclusion

This scoping review synthesized current evidence on advanced clinic-based technologies used to assess and monitor 3 key physical functions, namely balance, muscle strength, and ROM, in individuals surviving breast cancer. The review found that balance assessments predominantly utilized force plates and SOT, muscle strength was assessed using various models of the Biodex isokinetic dynamometer, and range of motion was measured using digital inclinometers and MMC systems. These studies were conducted primarily in hospitals and specialized medical facilities, with participant samples spanning various treatment phases and survivorship stages. The review documented considerable heterogeneity in measurement approaches, clinical settings, and reporting practices across the included studies. These findings underline that a diverse toolkit is currently applied to measure physical function in breast cancer survivorship, but that variability in methods limits cross-study comparability. Mapping this landscape can help us prioritize targeted validation and implementation studies and inform the development of pragmatic guidance for selecting feasible, clinically useful technologies in rehabilitation practice.

## Appendix

Multimedia Appendix 1 Complete search strategy and related keywords.

Multimedia Appendix 2 PRISMA-ScR (referred Reporting Items for Systematic Reviews and Meta-Analyses extension for Scoping Reviews).

Multimedia Appendix 3 Responses to Reviewers comments.

## Abbreviations

AP: anterior-posterior
APTA: American Physical Therapy Association
CoP: center of pressure
EDGE: Evaluation Database to Guide Effectiveness
HHD: handheld dynamometer
ICF: International Classification of Functioning, Disability and Health
MeSH: Medical Subject Headings
ML: medial-lateral
MMC: markerless motion capture
mHealth: mobile health
PRISMA: Preferred Reporting Items for Systematic Reviews and Meta-Analyses
ROM: range of motion
RMS: root mean square
SOT: Sensory Organization Test

## Data Availability

All data supporting the findings of this scoping review are included within the article and its supplementary materials. As this study synthesizes information from previously published literature, no new primary data were generated.
