# Supplementary material for: Advanced Clinical-Based Technologies for Monitoring Physical Function in Breast Cancer Survivors: Scoping Review
Source: JMIR Cancer. 2026 Jan 14;12:e77894. doi: 10.2196/77894 (PMC12853085; doi:10.2196/77894)
Supplement: Multimedia Appendix 1 [file cancer_v12i1e77894_app1.docx]

Table A. Search strategy for MEDLINE OVID All <1946 to February 01, 2024>

| Step Search term |
| --- |
| 1 exp Breast Neoplasms/  2 Breast cancer.mp.  3 breast tumor*.mp.  4 breast tumour*.mp.  5 breast neoplasm*.mp.  6 breast malignan*.mp.  7 breast carcinoma*.mp.  8 breast metasta*.mp.  9 breast oncolog*.mp.  10 or/1-9  11 exp Physical Functional Performance/  12 exp Hand Strength/ or exp Muscle Strength/  13 exp Pliability/  14 exp Physical Endurance/ or exp Endurance Training/  15 exp Postural Balance/  16 exp Walk Test/  17 exp "ROM, Articular"/  18 exp Exercise Test/  19 ("physical perform*" or "physical function*" or "physical activit*" or strength or flexibil* or enduran* or balanc* or equilibrium or "semi tandem" or tandem* or Romberg or "sit to stand*" or gait or walk* or ambulat* or grip or mobilit* or posture* or locomotion or "ROM*" or "upper extremity assessment*" or "chair rise test" or "chair rising test" or "chair stand test" or "Rising from a Chair Test" or "time up and go" or TUG or "timed up and go").mp.  20 Biological Monitoring/  21 exp Body Composition/ or exp Body Mass Index/  22 exp Heart Rate/ or exp Oxygen Consumption/ or exp Mitochondria/  23 exp Gait Analysis/  24 exp Bone Density/  25 video recording/ or motion capture/  26 wearable electronic devices/ or fitness trackers/  27 exp Muscle Strength Dynamometer/  28 exp Virtual Reality/  29 exp Accelerometry/  30 exp Electromyography/  31 exp Augmented Reality/  32 exp Actigraphy/  33 exp Absorptiometry, Photon/  34 exp Spirometry/  35 exp Spectroscopy, Near-Infrared/  36 exp Photogrammetry/  37 exp Sphygmomanometers/  38 (video or "marker less" or markerless or marker-based or "marker based*" or "motion monitor" or optitrack or vicon or dynamometer or electrogoniometer or "computerized gait*" or pedometer* or "functional movement screen*" or "pressure mapping system*" or "force plate*" or Kinect or KinectV2 or leap-motion-controller or pose2sim or deeplabcut or deep-lab-cut or trazer or lidar or "Light Buzz" or "Intel Depth Camera*" or Accelerometer or "Inertial measurement unit*" or "Balance platform*" or "Video analysis software*" or "Wearable sensor*" or Electromyography or "Pressure Sensing Insole*" or "Body-worn Camera*" or "Smartphone Sensor*" or "Virtual reality" or "Augmented reality" or "Digital goniometer*" or "Biomechanics Software*" or Actigraphy or "Bioelectrical Impedance Analysis*" or "High-Speed Camera*" or "Bod Pod" or "Dual-Energy X-ray" or absorptiometry or spirometry or "Metabolic Rate Analyzer*" or "Near-Infrared Spectroscopy" or photogrammetry or sphygmomanometer).mp.  39 25 or 26 or 27 or 28 or 29 or 30 or 31 or 32 or 33 or 34 or 35 or 36 or 37 or 38  40 11 or 12 or 13 or 14 or 15 or 16 or 17 or 18 or 19 or 20 or 21 or 22 or 23 or 24  41 10 and 39 and 40 |

The search strategy included the following keywords: (breast cancer or breast tumor* or breast tumour* or breast neoplasm* or breast malignan* or breast carcinoma* or breast metasta* or breast oncolog*).mp. AND ("physical perform*" or "physical function*" or "physical activit*" or strength or flexibil* or enduran* or balanc* or equilibrium or "semi tandem" or tandem* or Romberg or "sit to stand*" or gait or walk* or ambulat* or grip or mobilit* or posture* or locomotion or "ROM*" or "upper extremity assessment*" or "chair rise test" or "chair rising test" or "chair stand test" or "Rising from a Chair Test" or "time up and go" or TUG or "timed up and go").mp. AND (video or "marker less" or markerless or marker-based or "marker based*" or "motion monitor" or optitrack or vicon or dynamometer or electrogoniometer or "computerized gait*" or pedometer* or "functional movement screen*" or "pressure mapping system*" or "force plate*" or Kinect or KinectV2 or leap-motion-controller or pose2sim or deeplabcut or deep-lab-cut or trazer or lidar or "Light Buzz" or "Intel Depth Camera*" or Accelerometer or "Inertial measurement unit*" or "Balance platform*" or "Video analysis software*" or "Wearable sensor*" or Electromyography or "Pressure Sensing Insole*" or "Body-worn Camera*" or "Smartphone Sensor*" or "Virtual reality" or "Augmented reality" or "Digital goniometer*" or "Biomechanics Software*" or Actigraphy or "Bioelectrical Impedance Analysis*" or "High-Speed Camera*" or "Bod Pod" or "Dual-Energy X-ray" or absorptiometry or spirometry or "Metabolic Rate Analyzer*" or "Near-Infrared Spectroscopy" or photogrammetry or sphygmomanometer).mp.

APPENDICES:

Syntaxes:

Ovid MEDLINE(R) ALL <1946 to February 01, 2024>

1 exp Breast Neoplasms/ 349781

2 Breast cancer.mp. 349509

3 breast tumor*.mp. 24194

4 breast tumour*.mp. 4215

5 breast neoplasm*.mp. 351268

6 breast malignan*.mp. 1805

7 breast carcinoma*.mp. 33696

8 breast metasta*.mp. 955

9 breast oncolog*.mp. 313

10 or/1-9 464049

11 exp Physical Functional Performance/ 4301

12 exp Hand Strength/ or exp Muscle Strength/ 46116

13 exp Pliability/ 4624

14 exp Physical Endurance/ or exp Endurance Training/ 37923

15 exp Postural Balance/ 28608

16 exp Walk Test/ 2615

17 exp "Range of Motion, Articular"/ 60944

18 exp Exercise Test/ 71623

19 ("physical perform*" or "physical function*" or "physical activit*" or strength or flexibil* or enduran* or balanc* or equilibrium or "semi tandem" or tandem* or Romberg or "sit to stand*" or gait or walk* or ambulat* or grip or mobilit* or posture* or locomotion or "range of motion*" or "upper extremity assessment*" or "chair rise test" or "chair rising test" or "chair stand test" or "Rising from a Chair Test" or "time up and go" or TUG or "timed up and go").mp. 2154383

20 Biological Monitoring/ 1240

21 exp Body Composition/ or exp Body Mass Index/ 199790

22 exp Heart Rate/ or exp Oxygen Consumption/ or exp Mitochondria/ 448722

23 exp Gait Analysis/ 1492

24 exp Bone Density/ 61770

25 video recording/ or motion capture/ 28040

26 wearable electronic devices/ or fitness trackers/ 9484

27 exp Muscle Strength Dynamometer/ 1989

28 exp Virtual Reality/ 6338

29 exp Accelerometry/ 12376

30 exp Electromyography/ 85398

31 exp Augmented Reality/ 1379

32 exp Actigraphy/ 4819

33 exp Absorptiometry, Photon/ 26277

34 exp Spirometry/ 23691

35 exp Spectroscopy, Near-Infrared/ 16299

36 exp Photogrammetry/ 3397

37 exp Sphygmomanometers/ 3380

38 (video or "marker less" or markerless or marker-based or "marker based*" or "motion monitor" or optitrack or vicon or dynamometer or electrogoniometer or "computerized gait*" or pedometer* or "functional movement screen*" or "pressure mapping system*" or "force plate*" or Kinect or KinectV2 or leap-motion-controller or pose2sim or deeplabcut or deep-lab-cut or trazer or lidar or "Light Buzz" or "Intel Depth Camera*" or Accelerometer or "Inertial measurement unit*" or "Balance platform*" or "Video analysis software*" or "Wearable sensor*" or Electromyography or "Pressure Sensing Insole*" or "Body-worn Camera*" or "Smartphone Sensor*" or "Virtual reality" or "Augmented reality" or "Digital goniometer*" or "Biomechanics Software*" or Actigraphy or "Bioelectrical Impedance Analysis*" or "High-Speed Camera*" or "Bod Pod" or "Dual-Energy X-ray" or absorptiometry or spirometry or "Metabolic Rate Analyzer*" or "Near-Infrared Spectroscopy" or photogrammetry or sphygmomanometer).mp. 461397

39 25 or 26 or 27 or 28 or 29 or 30 or 31 or 32 or 33 or 34 or 35 or 36 or 37 or 38 481198

40 11 or 12 or 13 or 14 or 15 or 16 or 17 or 18 or 19 or 20 or 21 or 22 or 23 or 24 2787294

41 10 and 39 and 40 775

Web of science 2024-02-02 results 821

"breast cancer" OR "breast tumor*" OR "breast tumour*" OR "breast neoplasm*" OR "breast malignan*" OR "breast carcinoma*" OR "breast metasta*" OR "breast oncolog*" (Topic) and "physical perform*" OR "exercise test" OR "physical function*" OR "physical activit*" OR strength OR flexibil* OR enduran* OR balanc* OR equilibrium OR "semi tandem" OR tandem* OR romberg OR "sit to stand*" OR gait OR walk* OR ambulat* OR grip OR mobilit* OR posture* OR locomotion OR "range of motion*" OR "upper extremity assessment*" OR "chair rise test" OR "chair rising test" OR "chair stand test" OR "Rising from a Chair Test" OR "time up and go" OR tug OR "timed up and go" OR pliability OR "biological Monitoring" OR "Body Composition" OR "oxygen consumption" OR "heart rate" OR "bone density" (Topic) and video OR "marker less" OR markerless OR marker-based OR "marker based*" OR "motion monitor" OR optitrack OR vicon OR dynamometer OR electrogoniometer OR "computerized gait*" OR pedometer* OR "functional movement screen*" OR "pressure mapping system*" OR "force plate*" OR kinect OR kinectv2 OR leap-motion-controller OR pose2sim OR deeplabcut OR deep-lab-cut OR trazer OR lidar OR "Light Buzz" OR "Intel Depth Camera*" OR accelerometer OR "Inertial measurement unit*" OR "Balance platform*" OR "Video analysis software*" OR "Wearable sensor*" OR electromyography OR "Pressure Sensing Insole*" OR "Body-worn Camera*" OR "Smartphone Sensor*" OR "Virtual reality" OR "Augmented reality" OR "Digital goniometer*" OR "Biomechanics Software*" OR actigraphy OR "Bioelectrical Impedance Analysis*" OR "High-Speed Camera*" OR "Bod Pod" OR "Dual-Energy X-ray" OR absorptiometry OR spirometry OR "Metabolic Rate Analyzer*" OR "Near-Infrared Spectroscopy" OR photogrammetry OR sphygmomanometer (Topic)

Scopus: 2024-02-02 results 1458

"breast cancer" OR "breast tumor*" OR "breast tumour*" OR "breast neoplasm*" OR "breast malignan*" OR "breast carcinoma*" OR "breast metasta*" OR "breast oncolog*"

"physical perform*" OR "exercise test" OR "physical function*" OR "physical activit*" OR strength OR flexibil* OR enduran* OR balanc* OR equilibrium OR "semi tandem" OR tandem* OR romberg OR "sit to stand*" OR gait OR walk* OR ambulat* OR grip OR mobilit* OR posture* OR locomotion OR "range of motion*" OR "upper extremity assessment*" OR "chair rise test" OR "chair rising test" OR "chair stand test" OR "Rising from a Chair Test" OR "time up and go" OR tug OR "timed up and go" OR pliability OR "biological Monitoring" OR "Body Composition" OR "oxygen consumption" OR "heart rate" OR "bone density"

video OR "marker less" OR markerless OR marker-based OR "marker based*" OR "motion monitor" OR optitrack OR vicon OR dynamometer OR electrogoniometer OR "computerized gait*" OR pedometer* OR "functional movement screen*" OR "pressure mapping system*" OR "force plate*" OR kinect OR kinectv2 OR leap-motion-controller OR pose2sim OR deeplabcut OR deep-lab-cut OR trazer OR lidar OR "Light Buzz" OR "Intel Depth Camera*" OR accelerometer OR "Inertial measurement unit*" OR "Balance platform*" OR "Video analysis software*" OR "Wearable sensor*" OR electromyography OR "Pressure Sensing Insole*" OR "Body-worn Camera*" OR "Smartphone Sensor*" OR "Virtual reality" OR "Augmented reality" OR "Digital goniometer*" OR "Biomechanics Software*" OR actigraphy OR "Bioelectrical Impedance Analysis*" OR "High-Speed Camera*" OR "Bod Pod" OR "Dual-Energy X-ray" OR absorptiometry OR spirometry OR "Metabolic Rate Analyzer*" OR "Near-Infrared Spectroscopy" OR photogrammetry OR sphygmomanometer

CINHAL 2024-02-02 results 464

S37((S4 OR S5 OR S6 OR S7 OR S8 OR S9 OR S10 OR S11 OR S12 OR S13 OR S14 OR S15 OR S16 OR S17) AND (S20 OR S21 OR S22 OR S23 OR S24 OR S25 OR S26 OR S27 OR S28 OR S29 OR S30 OR S31 OR S32 OR S33 OR S34 OR S35)) AND (S3 AND S35 AND S36)

Database - CINAHL Plus with Full TextDisplayS36(S4 OR S5 OR S6 OR S7 OR S8 OR S9 OR S10 OR S11 OR S12 OR S13 OR S14 OR S15 OR S16 OR S17) AND (S18 OR S20 OR S21 OR S22 OR S23 OR S24 OR S25 OR S26 OR S27 OR S28 OR S29 OR S30 OR S31 OR S32 OR S33 OR S34)

Database - CINAHL Plus with Full TextDisplayS35S4 OR S5 OR S6 OR S7 OR S8 OR S9 OR S10 OR S11 OR S12 OR S13 OR S14 OR S15 OR S16 OR S17

Database - CINAHL Plus with Full TextDisplayS34( video or "marker less" or markerless or marker-based or "marker based*" or "motion monitor" or optitrack or vicon or dynamometer or electrogoniometer or "computerized gait*" or pedometer* or "functional movement screen*" or "pressure mapping system*" or "force plate*" or Kinect or KinectV2 or leap-motion-controller or pose2sim or deeplabcut or deep-lab-cut or trazer or lidar or "Light Buzz" or "Intel Depth Camera*" or Accelerometer or "Inertial measurement unit*" or "Balance platform*" or "Video analysis software*" or "Wearable sensor*" or Electromyography or "Pressure Sensing Insole*" or "Body-worn Camera*" or "Smartphone Sensor*" or "Virtual reality" or "Augmented reality" or "Digital goniometer*" or "Biomechanics Software*" or Actigraphy or "Bioelectrical Impedance Analysis*" or "High-Speed Camera*" or "Bod Pod" or "Dual-Energy X-ray" or absorptiometry or spirometry or "Metabolic Rate Analyzer*" or "Near-Infrared Spectroscopy" or photogrammetry or sphygmomanometer )

Database - CINAHL Plus with Full TextDisplayS33(MH "Kinematics")

Database - CINAHL Plus with Full TextDisplayS32(MH "Pedometers")

Database - CINAHL Plus with Full TextDisplayS31(MH "Sphygmomanometers+")

Database - CINAHL Plus with Full TextDisplayS30(MH "Photogrammetry")

Database - CINAHL Plus with Full TextDisplayS29(MH "Spectroscopy, Near-Infrared")

Database - CINAHL Plus with Full TextDisplayS28(MH "Spirometry")

Database - CINAHL Plus with Full TextDisplayS27(MH "Absorptiometry, Photon")

Database - CINAHL Plus with Full TextDisplayS26(MH "Actigraphy")

Database - CINAHL Plus with Full TextDisplayS25(MH "Augmented Reality")

Database - CINAHL Plus with Full TextDisplayS24(MH "Electromyography")

Database - CINAHL Plus with Full TextDisplayS23(MH "Accelerometry+")

Database - CINAHL Plus with Full TextDisplayS22(MH "Virtual Reality+")

Database - CINAHL Plus with Full TextDisplayS21(MH "Dynamometry")

Database - CINAHL Plus with Full TextDisplayS20(MH "Fitness Trackers")

Database - CINAHL Plus with Full TextDisplayS19

Database - CINAHL Plus with Full TextDisplayS18(MM "Motion Capture") OR (MM "Motion Analysis Systems")

Database - CINAHL Plus with Full TextDisplayS17(MH "Bone Density")

Database - CINAHL Plus with Full TextDisplayS16(MH "Gait Analysis")

Database - CINAHL Plus with Full TextDisplayS15(MH "Exercise Test, Cardiopulmonary") OR (MH "Exercise Test, Muscular+")

Database - CINAHL Plus with Full TextDisplayS14(MH "Heart Rate+")

Database - CINAHL Plus with Full TextDisplayS13(MH "Oxygen Consumption+")

Database - CINAHL Plus with Full TextDisplayS12(MH "Body Composition+")

Database - CINAHL Plus with Full TextDisplayS11(MH "Range of Motion")

Database - CINAHL Plus with Full TextDisplayS10(MH "Walking+")

Database - CINAHL Plus with Full TextDisplayS9(MH "Balance, Postural+") OR (MM "Balance Training, Physical") OR (MM "Balance (Iowa NOC)")

Database - CINAHL Plus with Full TextDisplayS8(MH "Physical Endurance+")

Database - CINAHL Plus with Full TextDisplayS7(MH "Pliability") OR (MH "Exercise Therapy: Joint Mobility (Iowa NIC)")

Database - CINAHL Plus with Full TextDisplayS6(MH "Grip Strength") OR (MH "Muscle Strength+")

Database - CINAHL Plus with Full TextDisplayS5(MH "Physical Performance")

Database - CINAHL Plus with Full TextDisplayS4( "physical perform*" or "physical function*" or "physical activit*" or strength or flexibil* or enduran* or balanc* or equilibrium or "semi tandem" or tandem* or Romberg or "sit to stand*" or gait or walk* or ambulat* or grip or mobilit* or posture* or locomotion or "range of motion*" or "upper extremity assessment*" or "chair rise test" or "chair rising test" or "chair stand test" or "Rising from a Chair Test" or "time up and go" or TUG or "timed up and go" )

Database - CINAHL Plus with Full TextDisplayS3(S1 OR S2)

Database - CINAHL Plus with Full TextDisplayS2( breast cancer or breast tumor* or breast tumour* or breast neoplasm* or breast malignan* or breast carcinoma* or breast metasta* or breast oncolog* )

Database - CINAHL Plus with Full TextDisplayS1(MH "Breast Neoplasms+")

**Gray literature:**

Thesis and dissertation

TS=("breast cancer" OR "breast tumor*" OR "breast tumour*" OR "breast neoplasm*" OR "breast malignan*" OR "breast carcinoma*" OR "breast metasta*" OR "breast oncolog*")

and

TS=("physical perform*" OR "exercise test" OR "physical function*" OR "physical activit*" OR strength OR flexibil* OR enduran* OR balanc* OR equilibrium OR "semi tandem" OR tandem* OR romberg OR "sit to stand*" OR gait OR walk* OR ambulat* OR grip OR mobilit* OR posture* OR locomotion OR "range of motion*" OR "upper extremity assessment*" OR "chair rise test" OR "chair rising test" OR "chair stand test" OR "Rising from a Chair Test" OR "time up and go" OR tug OR "timed up and go" OR pliability OR "biological Monitoring" OR "Body Composition" OR "oxygen consumption" OR "heart rate" OR "bone density") and

TS=(video OR "marker less" OR markerless OR marker-based OR "marker based*" OR "motion monitor" OR optitrack OR vicon OR dynamometer OR electrogoniometer OR "computerized gait*" OR pedometer* OR "functional movement screen*" OR "pressure mapping system*" OR "force plate*" OR kinect OR kinectv2 OR leap-motion-controller OR pose2sim OR deeplabcut OR deep-lab-cut OR trazer OR lidar OR "Light Buzz" OR "Intel Depth Camera*" OR accelerometer OR "Inertial measurement unit*" OR "Balance platform*" OR "Video analysis software*" OR "Wearable sensor*" OR electromyography OR "Pressure Sensing Insole*" OR "Body-worn Camera*" OR "Smartphone Sensor*" OR "Virtual reality" OR "Augmented reality" OR "Digital goniometer*" OR "Biomechanics Software*" OR actigraphy OR "Bioelectrical Impedance Analysis*" OR "High-Speed Camera*" OR "Bod Pod" OR "Dual-Energy X-ray" OR absorptiometry OR spirometry OR "Metabolic Rate Analyzer*" OR "Near-Infrared Spectroscopy" OR photogrammetry OR sphygmomanometer)

https://www.webofscience.com/wos/pqdt/summary/159e07e2-6885-4c58-8ed3-e2ff7cdb81f1-0103512a04/relevance/1

Considering PhD THESIS + English language from 72 we came up with 39 after importing to Convince we had one duplication 38 screened none of them met the criteria.
