# Supplementary material for: Advanced Clinical-Based Technologies for Monitoring Physical Function in Breast Cancer Survivors: Scoping Review
Source: JMIR Cancer. 2026 Jan 14;12:e77894. doi: 10.2196/77894 (PMC12853085; doi:10.2196/77894)
Supplement: Multimedia Appendix 3 [file cancer_v12i1e77894_app3.docx]

EDITORIAL COMMENTS (Decision: D/major revision)

-------------------

- Prior to resubmission carefully review the manuscript to verify that there are no typos or grammatical errors and that the writing style is clear, concise and easy to follow for readers without a specialized background in the subject matter.

There are several sections where there is redundant or duplicative information which could be communicated more concisely.

To enhance the scientific rigor of the manuscript, authors should ensure the manuscript follows standard scientific reporting principles, maintaining a formal, academic tone.

Thank you for these helpful editorial comments — the manuscript has been carefully revised to correct typographical and grammatical errors, remove redundant material, and improve clarity, concision, and formal academic tone throughout; all specific reviewer points have also been addressed (changes visible in the tracked-change file). Word count: Abstract:386 Main paper: 4013

- Abstract should be robust and reflective of the manuscript overall (word limit is 450).

* Expand the methods to include data synthesis approach

A1 RESPONSE: Methods: A comprehensive literature search was conducted across MEDLINE, Scopus, CINAHL, and Web of Science databases, with no publication date restrictions. Eligible studies included adults with breast cancer assessed using advanced clinical-based technologies to monitor physical function. Screening and selection followed PRISMA guidelines. Data extraction captured study characteristics, participant demographics, technologies applied, and related outcome. Extracted data were organized in Covidence and synthesized descriptively to map the types of technologies, assessed functional domains, and application settings across studies.

* Please include more quantitative/numerical results in the abstract (sample size, P-values, odds ratios, etc.). For example, if applicable for the statistical analysis performed, some of the point estimates/effect sizes may not have indices of variance and precision: Include indices of variance and precision (e.g., SD, confidence intervals).

A2 RESPONSE: Results: Across the 17 included studies (n=719; age range 30–75 years), participants were predominantly female and largely drawn from stage 0–III breast cancer cohorts; one study reported a single male participant, and two studies did not specify participant sex. Eleven studies were published from 2017 onward. Technologies spanned balance platforms (force plates, Technobody-PK 200 WL, Sensory Organization Test (SOT); n=5), isokinetic dynamometry (Biodex systems; n=4), and ROM assessment via motion capture (n=3) or digital inclinometers (n=5). Sample sizes per study ranged from 20 to 100 participants (median = 43), and follow-up durations varied from one session to six months.

- Provide a more detailed background in the Introduction section to strengthen the rationale for this review, and cite more scholarly references -pertinent/related articles in the past 2-3 years could be cited.

A3 RESPONSE: Introduction

Breast cancer remains the most prevalent malignancy among women worldwide, with increasing survival rates due to advances in early detection and treatment [1]. Although breast cancer mortality has declined in recent decades, as survival rates improve, attention has shifted from survival alone to long-term recovery, quality of life, and function, with rehabilitation emerging as a central component of survivorship care [2–4]. Physical function, the ability to perform physical tasks that enable daily activities and participation, is an important domain in cancer survivorship and rehabilitation and is framed within the International Classification of Functioning, Disability and Health (ICF)[5]. Impairments in physical function after breast cancer treatment are commonly reported and include deficits in balance, muscle strength, and range of motion [6–8]. Women undergoing chemotherapy may experience up to a 25% loss in strength and joint dysfunction [9]. Moreover, individual surviving with cancer highlighted difficulties with balance and walking as the most common functional issues, with prevalence rates of %19 and %24 respectively [10]. These functional limitations have been associated with poorer health-related quality of life and reduced mobility in survivorship cohorts [11,12]. At the same time, impairments in gait and balance control have been documented as potential contributors to fall risk and reduced independence in everyday life among breast cancer survivors [13]. According to the literature on cancer survivorship, over half of individuals who have undergone cancer treatments encounter physical function impairments [14].

The APTA Oncology EDGE Task Force has provided evidence-based recommendations for standardized outcome measures in oncology rehabilitation. For balance assessment, the Task Force strongly supports the use of low-cost, performance-based tools such as the Fullerton Advanced Balance Scale, gait speed, Timed Up and Go, Five Times Sit-to-Stand, and the Balance Evaluation Systems Test all rated as reliable and clinically feasible for cancer survivors [15]. In contrast, computerized balance systems such as force plates and the Sensory Organization Test (SOT) have been less highly recommended due to limited clinical utility and high cost, despite growing evidence supporting their sensitivity in detecting subtle postural sway and vestibular deficits in individuals with cancer and chemotherapy-induced peripheral neuropathy [16]. For shoulder range of motion, the Task Force rated passive goniometry (score 4) as a recommended tool, while for muscle strength, handheld dynamometers (HHDs) (score 3) and manual muscle testing (score 2B) were endorsed as appropriate clinical measures [17]. Although these conventional methods remain the clinical standard, their limited sensitivity and responsiveness underscore the need for more objective, automated technologies capable of quantifying subtle changes in function[18,19].

Furthermore, a variety of technologies such as wearable sensors (accelerometers/pedometers), fitness-tracker and smartphone apps, and advanced motion-capture systems are increasingly used to quantify physical function in breast cancer survivorship [20–24], providing objective, high-resolution data [23,24]. However, most reported applications remain in research or specialized settings with limited protocol and outcome standardization [21,24].

Accordingly, this review aims to map and characterize the use of such advanced, clinically based technologies in assessing key physical functions, including balance, muscle strength, and range of motion (ROM), among individuals surviving breast cancer. For the purposes of this review, “advanced” is defined as instruments that provide automated, objective outputs beyond unaided observation or analogue readouts. “Clinic-based” denotes systems that can be operated in clinical rooms or clinically configured spaces by routine clinical staff with minimal specialist engineering support, for monitoring and assessing physical functions. These terms were selected based on their relevance within the ICF framework and their frequent association with impairment among breast cancer survivors [13,25,26]. By examining how these technologies are currently integrated into clinical practice, this review seeks to identify gaps in the existing literature and highlight areas where further research is needed.

- There are reporting shortcomings in the methods. Please ensure adequate details are provided to facilitate reproducibility of all study related procedures and analysis. This includes more information about:

*Expand how the search strategy was developed. Was a librarian or information scientist consulted? What informed the selection of terms?

A4 RESPONSE: The initial search strategy was developed in collaboration with an experienced academic health sciences librarian (Liz Dennett) to ensure methodological rigour and comprehensive coverage. The selection of search terms was informed by preliminary scoping searches and key indexing terms from prior reviews in oncology rehabilitation and motion analysis. The MEDLINE strategy served as the base and was adapted for syntax variations across databases.

*How were duplicates handled?

A5 RESPONSE: Duplicate records were automatically identified and removed by Covidence’s built-in algorithm, followed by manual verification by the reviewers to ensure accuracy.

* The flow chart reports grey literature, masters these and citation searching but I do not see this reflected in the methods.

A6 RESPONSE: In addition to peer-reviewed databases, grey-literature searches were limited to PhD dissertations. All identified articles were imported into Covidence for screening.

* The paragraph describing synthesis is descriptive but it remains unclear the procedures that were completed and how the reviewers approached synthesis.

A7 RESPONSE: The extracted data from relevant studies were synthesized narratively and summarized in tabular format to provide a comprehensive overview of advanced clinical-based technologies used to monitor and assess functional outcomes in individuals surviving breast cancer. Studies were first categorized by the component of body function assessed (balance, strength, and ROM) and then organized according to the specific measurement technology employed. For each technology identified, the corresponding data analysis methods used in the original studies were extracted and documented. This synthesis sought to deliver an analysis of the current knowledge and methodologies used to assess the included physical functions. The findings were analyzed to highlight common methodologies, measurement tools, and outcomes reported in the literature. This process defined how functional outcomes are commonly evaluated in individuals with breast cancer using advanced and clinical-based technologies and identify potential gaps or inconsistencies in current practices, including variations in measurement protocols, differences in data normalization methods, and the range of technologies applied to assess similar functional outcomes.

- Currently the results is a narrative summary of the studies and not a synthesis of the evidence. Please revise and include more quantitative data - i.e. specify the number of studies

A8 RESPONSE Characteristics of included studies

A review of 17 studies focusing on individuals surviving breast cancer revealed key insights into research locations, designs, timelines, participant stages, and objectives. The United States emerged as the most common location, hosting seven of the included studies. The remaining research was conducted across a diverse set of countries, including Finland, Turkey, Portugal, Belgium, Brazil, South Korea, India, and Denmark. The review included five experimental studies: three randomized controlled trials [28,33,38], one pretest– post-test experimental study [29], and one pilot intervention; [44] and twelve observational studies: four cross-sectional studies [32,34,35,40], three case-control studies [36,42,43], two prospective observational studies [30,31], two longitudinal cohort studies [37,39], and one descriptive study [41]. The publication timeline indicates a growing interest in this area, with 11 of the 17 studies published in 2017 or later, while the remaining five studies were published between 2003 and 2016 [39–44]. The included studies sampled individuals across treatment and survivorship phases, with participants’ ages ranging roughly from the early 30s to mid-70s and most cohorts drawn from stage 0–III breast cancer. Study samples included people receiving hormone therapy, newly diagnosed patients, individuals undergoing or recently completing chemotherapy, women evaluated after reconstructive surgery, and postmenopausal survivors. Among the 15 studies that reported participant sex, all included only female participants except for one study, which included a single male participant [37]. Two studies did not report any information about participant sex. Time since treatment and stage reporting varied across studies, reflecting the heterogeneity of the reviewed samples. The studies aim to assess different physical functions, with a particular emphasis on balance, strength, flexibility, and functional impairments (e.g., fatigue) related to breast cancer treatments. The objectives of the studies were varied, ranging from evaluating the effectiveness of specific interventions, such as Mat Pilates and myofascial techniques, to assessing the impact of cancer-related fatigue on balance and postural control. Some studies also investigate the feasibility of motion capture technologies for screening and assessing functional impairments.

Furthermore, the studies included in the review were conducted in multiple settings, predominantly within specialized medical facilities. Many studies took place in hospitals or hospital-affiliated centers; within these settings, force plates and the SOT were reported almost exclusively. Isokinetic dynamometry using Biodex was reported mainly in hospital settings [28,30], with one study conducted in a laboratory environment [33]. Digital inclinometers were used across hospitals, rehabilitation services, and outpatient oncology clinics [36,38,39,43]. Kinect-based markerless motion capture (MMC) was implemented within oncology services spanning a medical center, and an outpatient oncology clinic [35,40,41]. One study even extended its setting to participants’ homes or convenient community sites [44].

In terms of recruitment strategies, many studies enrolled participants through state and hospital cancer registries, clinician referrals, and ongoing treatment facilities within large urban hospitals. Researchers also leveraged community outreach by utilizing social media platforms, distributing flyers in regional cancer hospitals, and engaging with breast cancer support groups. Local media outlets, such as newspapers and radio announcements, were employed to broaden their reach. Some studies relied on word-of-mouth, emails, and telephone contacts.

The studies included in this review primarily focused on individuals surviving breast cancer, with participants aged between 30 and 75 years, and an average age range of 50 to 60 years. Nine of the 17 included studies (53%) explicitly reported participants' cancer stage; all nine enrolled individuals with stage 0–III disease [28,32–34,37,41–44]. Five studies involved participants undergoing or having recently completed chemotherapy [29,32–34,43], and one study specifically enrolled women receiving hormone therapy [28]. One study focused on surgical reconstruction (LD flap) [30]. Two studies included post-mastectomy survivors compared to healthy controls [31,34], and three enrolled mixed-treatment cohorts [29,32,42]. In terms of sample sizes, these varied considerably across the studies, ranging from as few as 20 participants to as many as 100. People with stage IV cancer and cognitive impairments were generally excluded. Additional exclusion criteria, such as uncontrolled cardiovascular or musculoskeletal conditions, severe neuropathy, or inability to provide informed consent, were applied to ensure participant safety and maintain the validity of assessment results.

Reported Instruments and Key Findings on Physical Function

This scoping review found that most studies assessed physical function using a variety of outcome measures, ROM was reported in 8 of the 17 included studies (47.1%) [29,35,36,38–41,43]. Balance outcomes were reported in 5 studies (29.4%) [31,32,34,37,42], and muscle strength outcomes were reported in 4 studies (23.5%) [28,30,33,44]. Of these, three studies employed motion capture technologies [35,40,41], Kinect-based systems to assess upper extremity kinematics and ROM in individuals surviving breast cancer. Inclinometers were used in five studies to further quantify shoulder movements [29,36,38,39,43]. Balance assessments were another key focus, with multiple studies using advanced tools to evaluate postural stability and fall risk. Two studies employed force plates to measure postural sway in both static and dynamic conditions [32,37], while the SOT was used in another two studies to assess balance under varying sensory inputs [34,42].

Additionally, strength assessments were conducted using different models of the Biodex System (2, 3, 4, and 4 Pro) to evaluate isometric and isokinetic strength in muscle groups including the shoulder, hip, knee, and wrist [28,30,33,44]. Measurements included isometric strength normalized to body weight, as well as peak torque during isokinetic contractions at varying angles and velocities. Further details on the specific tools and data analysis methods used in each study are provided in Table 2.

- Authors are advised to more carefully distinguish Methods, Results, and Discussion throughout the manuscript. In particularly, carefully separate Methods from Results. For instance, "The initial search yielded 3,518 references" is a result but is reported in the methods.

A9 RESPONSE: We appreciate your valuable comment, we have carefully revised the manuscript to ensure clear separation of Methods, Results, and Discussion, and all related comments have been addressed.

- "The review also included three cross-sectional studies, three case-control studies, two prospective studies, two longitudinal studies, one descriptive study, and one observational study. "

This is confusing as these don’t seem to be mutually exclusive study designs – what is the difference between prospective and longitudinal? What is the difference between descriptive and observational?

A10 RESPONSE: The review included five experimental studies: three randomized controlled trials [28,33,38], one pretest–post-test experimental study [29], and one pilot intervention; [44] and twelve observational studies: four cross-sectional studies [32,34,35,40], three case-control studies [36,42,43], two prospective observational studies [30,31], two longitudinal cohort studies [37,39], and one descriptive study [41].

Note: In our review, the two prospective observational studies followed participants from a defined starting point to observe outcomes as they occurred, while the two longitudinal cohort studies involved repeated assessments over time to monitor changes within the same participants.

- Discussion should be structured as follows:

1. Brief summary of the main study findings relative to the stated Introduction objectives/aims.

A11 RESPONSE: This scoping review mapped 17 studies deploying advanced clinic-based technologies to assess physical function in breast cancer survivors. The review identified technologies used to assess three domains, ROM, balance and muscle strength, and documented variation in instrument type. These technologies were applied in varied clinical environments, primarily hospital-based and outpatient oncology settings, to measure specific aspects of physical function through structured protocols.

2. Detailed discussion of those findings, including interpretations, implications, and comparisons to existing literature. 3. Discussion of limitations.

A12 RESPONSE: Studies that used advanced, clinic-based balance technologies, instrumented assessments targeted different aspects of postural control and consistently yielded information that simple, single-condition tests may miss. SOT protocols revealed impairments under proprioceptive and vestibular challenging conditions, despite near-normal performance on easier conditions, indicating that breast cancer survivorship related balance deficits can be context-dependent rather than global [34,42]. This pattern supports the clinical value of multi-condition batteries that probe sensory reweighting, rather than relying solely on single-task screens. Force plates quantify quiet-stance stability by deriving center of pressure (CoP) signals and summarizing them with standard sway metrics, typically medial–lateral (ML) and anterior–posterior (AP) excursion (range or root mean square (RMS)), total path length, mean sway velocity, and planar sway area (often a 95% confidence ellipse). These metrics capture both the magnitude (e.g., excursion, area) and temporal dynamics (e.g., velocity) of postural control. Notably, key ML CoP metrics including mean velocity, mean amplitude , and RMS displacement, have been linked to higher prospective fall risk [45]. In the included longitudinal study of taxane-based chemotherapy, Monfort et al. [37] used a laboratory force plate to track changes in CoP behavior during treatment; cumulative taxane exposure was associated with deteriorations in balance control, reflected by increased sway and concurrent gait alterations (shorter step length, slower walking speed). Complementing this, Wechsler et al. [32] showed that cancer-related fatigue independently predicted poorer postural stability on force-plate measures, consistent with compensatory stabilization strategies and greater sway under static and dynamic conditions. Together, these findings suggest that when clinically relevant stressors are present (neurotoxic chemotherapy, fatigue), force-plate CoP metrics are sensitive to subtle balance changes.

For ROM measurement the review documents a technology continuum from operator-dependent digital inclinometers widely feasible but with persistent measurement limitations to automated MMC systems offering three-dimensional, bias-reduced assessment with emerging evidence of clinical utility. The successful application of MMC for upper extremity ROM assessment in breast cancer survivors, demonstrated across three studies in diverse settings including oncology clinics and outpatient centers, suggests these technologies have overcome initial feasibility barriers and may be ready for broader clinical implementation[35,40,41]. The strong correlation between Kinect-derived reachable workspace metrics and QuickDASH, a validated and widely used tool for assessing upper extremity disability in this population [46], while the system's ability to detect side-to-side differences in functionally relevant movement zones (overhead reaching) demonstrates practical utility for monitoring recovery[35]. Nonetheless, substantial heterogeneity in measurement protocols, encompassing whether motion is assessed actively versus passively, the specific planes or tasks evaluated, and the choice of absolute versus side-to-side metrics, complicates cross-study synthesis and limits the development of coherent, evidence-based clinical guidelines. Taken together, within the bounds of the included studies, MMC appears to have promising potential for assessing upper-extremity function in breast cancer survivors.

Muscle strength was quantified with isokinetic dynamometry in controlled clinical or laboratory settings [28,30,33,44]. Systems such as the Biodex (models 2–4/4 Pro) are widely regarded as criterion instruments for dynamic muscle assessment because they impose precise, preset angular velocities and capture full torque–angle–velocity relationships together with work and power outputs. These multidimensional profiles can reveal deficits in force production and velocity-dependent behavior (e.g., concentric vs. eccentric weakness) that are not apparent from single-value manual grades, and their test–retest reliability in musculoskeletal [47,48]. The trade-off is practical, true isokinetic testing requires dedicated equipment, space, regular calibration, and staff skilled in positioning, stabilization, familiarization, and protocol standardization, all of which limit routine deployment outside well-resourced centers. Given these constraints, many rehabilitation services rely on portable HHDs for strength assessment. Professional guidance from the APTA Oncology EDGE Task Force recognizes HHDs as an appropriate option for cancer populations, provided that clinicians use standardized patient and tester positioning, consistent lever arms, and repeated trials to improve reliability [17].

This scoping review had some limitations. First, the inclusion of solely English-language studies raised the possibility of language bias and the exclusion of relevant work that had been published in other languages. Additionally, the review focused solely on those three physical functions, potentially overlooking other important aspects of physical function in individuals surviving breast cancer. While multiple databases were searched, some relevant studies may not have been captured if they were published in databases that were not included in the search strategy which can limit the comprehensiveness of the review. Finally, the variability of included studies in in design, clinical setting, treatment phase, and outcome definitions (e.g., active vs passive ROM, task/plane selection, absolute vs interlimb metrics), which precluded quantitative synthesis and limits cross-study comparability. Specifically, individuals with stage IV cancer were typically excluded in most of the included studies to focus on non-metastatic cases.

4. Conclusions that don’t just summarise the findings, but speak to the broader implications.

* Ensure that all statements in the Discussion and Conclusions appropriately match the methodology and results (applies to the Abstract and the manuscript text).

A13 RESPONSE: Conclusion

This scoping review synthesized current evidence on advanced clinical-based technologies used to assess and monitor of key physical functions: balance, muscle strength, and ROM in individuals surviving breast cancer. The review found that balance assessments predominantly utilized force plates and the SOT, muscle strength was assessed using various models of the Biodex isokinetic dynamometer, and range of motion was measured using digital inclinometers and MMC systems. These studies were conducted primarily in hospital and specialized medical facilities, with participant samples spanning various treatment phases and survivorship stages. The review documented considerable heterogeneity in measurement approaches, clinical settings, and reporting practices across the included studies. These findings underline that a diverse toolkit is currently applied to measure physical function in breast cancer survivorship but that variability in methods limits cross-study comparability. Mapping this landscape can help prioritize targeted validation and implementation studies and inform the development of pragmatic guidance for selecting feasible, clinically useful technologies in rehabilitation practice.

- Please see this information about particular figure types which are not permitted by JMIR Publications' journal style and revise your figures accordingly: https://support.jmir.org/hc/en-us/articles/360013033312-What-types-of-figures-are-not-permitted-in-JMIR-articles

- Table 1 can be moved to the supplemental file with the search strategy in the other databases- Table 2 and Figure 2 can be removed as the information can be presented in the text.

A14 RESPONSE: Table 1 Removed to supplementary materials. Table 2 & Figure2 their information presented in the text.

- Please correct Figure 1

Is it correct that 292 articles were not retrieved?

It is incorrect that 17 full texts were assessed for eligibility and that 2035 were excluded at this stage. Please fix.

A15 RESPONSE: All comments applied in the diagram.

- All in-text references must be numbers in square brackets. Do not use the author-year system, round brackets, or superscripts. Follow our instructions for authors for references: https://support.jmir.org/hc/en-us/articles/115001333067-How-should-references-be-formatted-Which-journal-style-should-I-choose-when-using-Endnote-

- Author Contributions - Please add a statement of Author Contributions according to the CRediT taxonomy. https://credit.niso.org/

A16 RESPONSE:

Author Contributions

Conceptualization: Mahtab Azhdar (MA), Martin Ferguson-Pell (MFP), Adalberto Loyola-Sanchez (ALS)

Methodology: MA, MFP, Amber Wardrop (AW)

Formal analysis: MA, MFP

Investigation: MA, MFP

Data curation: MA, MFP, AW

Writing – original draft: MA, MFP

Writing – review & editing: MA, MFP, ALS

Visualization: MA

Supervision: MFP, ALS

Project administration: MA, MFP

- Funding Statement - Add a funding statement, distinct from the Acknowledgements, even if no funding was involved. More information: https://support.jmir.org/hc/en-us/articles/360015982471-How-should-the-Acknowledgments-section-be-formatted-

A17 RESPONSE: Funding: No funding was received for this research.

- Disclose whether generative AI was used in any portion of the manuscript generation. If none, authors should attest to this in the response to comments. https://support.jmir.org/hc/en-us/articles/13387268671771-Do-you-allow-the-the-use-of-ChatGPT-or-other-generative-language-models-and-how-should-this-be-reported-

A18 RESPONSE: Text editing and language polishing were assisted using ChatGPT by OpenAI*. All content was reviewed and approved by the authors.

*Instructions*

Please upload a revised version with tracked changes (or highlighted sections of text where changes were made) as a supplementary file, in addition to providing a clean version under "Revised Ms". See https://jmir.zendesk.com/hc/en-us/articles/115001400448 for details on how to respond to reviewer comments and upload a revised manuscript. Supplying a version of the manuscript with tracked changes facilitates the review process.

Note that it is the authors' responsibility to ensure adherence to JMIR Publications style and formatting, including final word count.

Addressing formatting issues and any remaining comments during the revisions period speeds up the production process in the event of acceptance.

### Further Editorial / Peer-reviewer Comments:

---------- snip ----------

------------------------------------------------------

Reviewer BQ:

General comments

=============

The present review gives a literature based analysis on the use of advanced clinical-based technologies for measuring specific body functions (balance, range of motion, and strength) in individuals surviving breast cancer. An overview of the use of these technologies is applied in diverse samples of individuals surviving breast cancer. This gives inside in the current trends of use of objective measurement for specific body function. The auhors conculdes that the review reveals areas where further research is needed to improve the assessment of physical function in this group. Although interesting inside in the current use of advanced technologies for measurement of specific body function is given the review shows limitations which are given in the specific comments.

Specific comments

=============

Major comments

---------------------

1. I would be helpful the setting of use of this advanced technology is more included in the analyses.

B1 RESPONSE: Furthermore, the studies included in the review were conducted in multiple settings, predominantly within specialized medical facilities. Many studies took place in hospitals or hospital-affiliated centers; within these settings, force plates and the SOT were reported almost exclusively. Isokinetic dynamometry using Biodex was reported mainly in hospital settings [28,30], with one study conducted in a laboratory environment [33]. Digital inclinometers were used across hospitals, rehabilitation services, and outpatient oncology clinics [36,38,39,43]. Kinect-based markerless motion capture (MMC) was implemented within oncology services spanning a medical center, and an outpatient oncology clinic [35,40,41]. One study even extended its setting to participants’ homes or convenient community sites [44].

2. The review focus to specific body functions not to all functions relevant for physical activity monitoring that should be better given. The title could be better focused.

B2 RESPONSE: Thank you for this thoughtful comment. We agree that our review focuses on specific domains within physical function. To clarify this scope while maintaining terminology consistent with rehabilitation practice, we now state explicitly, beginning in the Abstract and Introduction and reiterated in Methods, that our synthesis is limited to balance, muscle strength, and range of motion (ROM) as key domains of physical function in breast cancer survivors. We have retained the umbrella term physical function for conceptual coherence and alignment with contemporary rehabilitation literature, and we have refined the title and keywords to name these three domains explicitly.

3. The exclusion criteria for lab-based capture motion systems should be more detailed described.

B3 RESPONSE: To preserve methodological rigor while ensuring a clinically actionable synthesis, an additional consultation with the librarian was conducted to refine the eligibility criteria prior to initiating full-text review and data extraction. The inclusion criteria were subsequently refined to prioritize advanced, clinically deployable technologies for point-of-care assessment of three core domains of physical function: balance, muscle strength, and range of motion. For the purposes of this review, “advanced” is defined as instruments that provide automated, objective outputs beyond unaided observation or analogue readouts, and “clinic-based” denotes systems that can be operated in clinical rooms or clinically configured spaces by routine clinical staff with minimal specialist engineering support. This refinement also served to avoid redundancy with existing systematic reviews and meta-analyses focused on accelerometer and pedometer studies.

4. How was the decision process between the three reviewers should be described in more detail.

B4 RESPONSE: The study selection process was conducted in several stages following established systematic review guidelines. Initially, two reviewers (M.A. and A.W.) independently screened titles and abstracts of all retrieved references against the eligibility criteria. Discrepancies between the two reviewers at this stage were flagged within Covidence for subsequent resolution. Studies deemed potentially eligible by either reviewer advanced to full-text screening, which was similarly conducted independently by the same two reviewers (M.A. and A.W.). Following both screening phases, conflicts were systematically resolved through a structured consensus process. For any study where the two primary reviewers disagreed on inclusion or exclusion, the full text and relevant eligibility criteria were reviewed collaboratively by all three reviewers (M.A., A.W., and M.F.P.) in a consensus meeting. During these discussions, each reviewer presented their rationale, and discrepancies were resolved through deliberation until unanimous agreement was reached. If consensus could not be achieved through discussion, the third reviewer (M.F.P.) served as the final arbiter. All final inclusion decisions and data extraction were completed through this consensus process, with regular meetings held among the three reviewers to ensure consistency in interpretation of eligibility criteria and data extraction procedures throughout the review.

5. More focus to the requirements for confirmation of validity, reliability and clinical utility of the advanced technology in the given use case is necessary.

B5 RESPONSE Thank you for this valuable comment. We agree that evaluating the validity, reliability, and clinical utility of advanced technologies is an important direction for future research. However, the primary objective of this scoping review was to map and synthesize the current use of advanced clinical-based technologies in assessing physical functions among individuals surviving breast cancer, rather than to critically appraise or compare measurement properties. Therefore, while we acknowledge the importance of psychometric evaluation, it was beyond the scope of the present review’s research question and methodological framework.

2.

3.

...

Minor comments

---------------------

4. ...

------------------------------------------------------

Reviewer GJ:

General comments

=============

Your review appears to follow the PRISMA guidelines for scoping reviews, but it’s difficult to follow and your Results section lacks detail. PRISMA does seem to recommend summarizing results in the Discussion section but you still need to report them in some detail in your Results section. Please consider the following comments and suggestions.

Specific comments

=============

Major comments

---------------------

1.Abstract

Background – You describe the rationale for your review as conventional assessments lack precision and real-time feedback, but I can’t find any evidence to support that in your Introduction. Your assertion that technologies are better is not the same as saying that the traditional assessments aren’t precise, etc.

C1 RESPONSE: Revised Background: Individuals surviving breast cancer often face long-term impairments in physical function, significantly impacting their quality of life. In recent years, a variety of technologies have been developed to monitor and assess these functions; however, there is no consolidated synthesis linking specific technologies to targeted functional domains and real-world clinical contexts, limiting comparability and translation into practice.

Methods – Please add the publication date parameters for your search. Also, please clarify what you mean by “key findings.” Weren’t the technologies your key findings?

C2 RESPONSE: This statement is added in abstract and manuscript “no publication date restrictions were applied.”

In our data extraction table, the “Key Finding” column refers to the main outcome reported by each included study regarding the physical function assessed using the specified technology. Revised version” Data extraction captured study characteristics, participant demographics, technologies used, and key findings related to the technology’s application.” Also, in the method section “data extraction” “…and key findings reported by the original authors in relation to the use or outcomes of the measurement technology.”

Results – In addition to participant age, please add gender or sex to your results. Were all participants female or were there studies with male breast cancer survivors? Be more precise than just saying that inclinometers “dominated.” How many studies or what percentage used inclinometers? How did you determine “promising reliability and portability” for markerless systems? Reliability and portability don’t seem consistent with your objective. The results you’re describing regarding Biodex systems seem to be more of a conclusion (your opinion) than data. For example, I don’t see data regarding cost in your results, yet you conclude that Biodex are precise but costly.

C3 RESPONSE: Sex & gender in abstract: Across the 17 included studies (n=719; age range 30–75 years), participants were predominantly female and largely drawn from stage 0–III breast cancer cohorts; one study reported a single male participant, and three studies did not specify participant sex.

Sex &gender in the main text: The included studies sampled individuals across treatment and survivorship phases, with participants’ ages ranging roughly from the early 30s to mid-70s and most cohorts drawn from stage 0–III breast cancer. Study samples included people receiving hormone therapy, newly diagnosed patients, individuals undergoing or recently completing chemotherapy, women evaluated after reconstructive surgery, and postmenopausal survivors. Among the 15 studies that reported participant sex, all included only female participants except for one study, which included a single male participant [37]. Two studies did not report any information about participant sex.

How many studies or what percentage used inclinometers This scoping review found that most studies assessed physical function using a variety of outcome measures, ROM was reported in 8 of the 17 included studies (47.1%) [29,35,36,38–41,43]. Balance outcomes were reported in 5 studies (29.4%) [31,32,34,37,42], and muscle strength outcomes were reported in 4 studies (23.5%) [28,30,33,44]. Of these, three studies employed motion capture technologies [35,40,41], Kinect-based systems to assess upper extremity kinematics and ROM in individuals surviving breast cancer. Inclinometers were used in five studies to further quantify shoulder movements [29,36,38,39,43].

Thank you for this valuable comment. The Results section was revised to ensure that only data-derived findings are presented. The interpretive statements regarding the reliability and portability of markerless systems and the cost of Biodex equipment were removed from the Results.

Conclusion – Use of “essential” doesn’t seem to be supported by your study findings. You have no evidence regarding the essentialness of standardization in order to broaden clinical adoption.

C4 RESPONSE: Revised conclusion: This scoping review synthesized current evidence on advanced clinic-based technologies used to assess and monitor of key physical functions: balance, muscle strength, and range of motion in individuals surviving breast cancer. The review found that balance assessments predominantly utilized force plates and the SOT, muscle strength was assessed using various models of the Biodex isokinetic dynamometer, and range of motion was measured using digital inclinometers and MMC systems. These studies were conducted primarily in hospital and specialized medical facilities, with participant samples spanning various treatment phases and survivorship stages. The review documented considerable heterogeneity in measurement approaches, clinical settings, and reporting practices across the included studies. These findings underline that a diverse toolkit is currently applied to measure physical function in breast cancer survivorship but that variability in methods limits cross-study comparability. Mapping this landscape can help prioritize targeted validation and implementation studies and inform the development of pragmatic guidance for selecting feasible, clinically useful technologies in rehabilitation practice.

2.Introduction

The Introduction should be a factual, objective review of the evidence. Use of terms such as “critical” and “crucial” aren’t supported by evidence and therefore, are not appropriate. They give your Introduction the quality of a convincing essay rather than a review of the literature. Please revise your Introduction to provide evidence-based statements supported by original study results. For example, your use of Diaz-Buxo et al. (Reference #4) doesn’t seem to support your statement about physical function predicting mortality and morbidity. Diaz-Buxo et al. evaluated differences in quality of life using the SF-36 between different groups of dialysis patients. They didn’t evaluate the effect of function on mortality or morbidity. I have the same concern regarding your use of Ferlay et al. (Reference #7) to support your statement that impairments influence quality of life. The study only reported numbers of cancer cases. It did not evaluate or report changes in quality of life and did not discuss function. These are only two examples and not an exhaustive list. Please go through and review all of your sources to ensure they are accurate and appropriate to support your statements.

C5 RESPONSE: Revised introduction: Breast cancer remains the most prevalent malignancy among women worldwide, with increasing survival rates due to advances in early detection and treatment [1]. Although breast cancer mortality has declined in recent decades, as survival rates improve, attention has shifted from survival alone to long-term recovery, quality of life, and function, with rehabilitation emerging as a central component of survivorship care [2–4]. Physical function, the ability to perform physical tasks that enable daily activities and participation, is an important domain in cancer survivorship and rehabilitation and is framed within the International Classification of Functioning, Disability and Health (ICF)[5]. Impairments in physical function after breast cancer treatment are commonly reported and include deficits in balance, muscle strength, and range of motion [6–8]. Women undergoing chemotherapy may experience up to a 25% loss in strength and joint dysfunction [9]. Moreover, individual surviving with cancer highlighted difficulties with balance and walking as the most common functional issues, with prevalence rates of %19 and %24 respectively [10]. These functional limitations have been associated with poorer health-related quality of life and reduced mobility in survivorship cohorts [11,12]. At the same time, impairments in gait and balance control have been documented as potential contributors to fall risk and reduced independence in everyday life among breast cancer survivors [13]. According to the literature on cancer survivorship, over half of individuals who have undergone cancer treatments encounter physical function impairments [14].

The APTA Oncology EDGE Task Force has provided evidence-based recommendations for standardized outcome measures in oncology rehabilitation. For balance assessment, the Task Force strongly supports the use of low-cost, performance-based tools such as the Fullerton Advanced Balance Scale, gait speed, Timed Up and Go, Five Times Sit-to-Stand, and the Balance Evaluation Systems Test all rated as reliable and clinically feasible for cancer survivors [15]. In contrast, computerized balance systems such as force plates and the Sensory Organization Test (SOT) have been less highly recommended due to limited clinical utility and high cost, despite growing evidence supporting their sensitivity in detecting subtle postural sway and vestibular deficits in individuals with cancer and chemotherapy-induced peripheral neuropathy [16]. For shoulder range of motion, the Task Force rated passive goniometry (score 4) as a recommended tool, while for muscle strength, handheld dynamometers (HHDs) (score 3) and manual muscle testing (score 2B) were endorsed as appropriate clinical measures [17]. Although these conventional methods remain the clinical standard, their limited sensitivity and responsiveness underscore the need for more objective, automated technologies capable of quantifying subtle changes in function[18,19].

Furthermore, a variety of technologies such as wearable sensors (accelerometers/pedometers), fitness-tracker and smartphone apps, and advanced motion-capture systems are increasingly used to quantify physical function in breast cancer survivorship [20–24], providing objective, high-resolution data [23,24]. However, most reported applications remain in research or specialized settings with limited protocol and outcome standardization [21,24].

Accordingly, this review aims to map and characterize the use of such advanced, clinically based technologies in assessing key physical functions, including balance, muscle strength, and range of motion (ROM), among individuals surviving breast cancer. For the purposes of this review, “advanced” is defined as instruments that provide automated, objective outputs beyond unaided observation or analogue readouts. “Clinic-based” denotes systems that can be operated in clinical rooms or clinically configured spaces by routine clinical staff with minimal specialist engineering support, for monitoring and assessing physical functions. These terms were selected based on their relevance within the ICF framework and their frequent association with impairment among breast cancer survivors [13,25,26]. By examining how these technologies are currently integrated into clinical practice, this review seeks to identify gaps in the existing literature and highlight areas where further research is needed.

This is a minor point but I suggest standardizing your terminology regarding function. “Body functions” refer to physiological functions such as metabolism, respiration, and excretion. Perhaps consistent use of “physical function” would be more meaningful and consistent with the point of your review.

C6 RESPONSE: Thank you for this suggestion. We have standardized terminology to physical function throughout the manuscript for clarity and consistency with common usage in rehabilitation oncology. We retain a brief note in the Introduction linking our targeted domains (balance, strength, ROM) to their underlying ICF body function components to preserve conceptual alignment.

Aims statement (page 5) – To ensure that readers understand your study purpose, I recommend moving the definition of “clinical-based technologies” from page 10 to your Introduction, or at least providing an adequate explanation of what they are rather than just identifying the term.

C7 RESPONSE: Thanks for your great comment, it is applied. Accordingly, this review aims to map and characterize the use of such advanced, clinically based technologies in assessing key physical functions, including balance, muscle strength, and range of motion (ROM), among individuals surviving breast cancer. For the purposes of this review, “advanced” is defined as instruments that provide automated, objective outputs beyond unaided observation or analogue readouts. “Clinic-based” denotes systems that can be operated in clinical rooms or clinically configured spaces by routine clinical staff with minimal specialist engineering support, for monitoring and assessing physical functions. These terms were selected based on their relevance within the ICF framework and their frequent association with impairment among breast cancer survivors [13,25,26]. By examining how these technologies are currently integrated into clinical practice, this review seeks to identify gaps in the existing literature and highlight areas where further research is needed.

3.Method

Was your search data limited? What parameters did you include for publication date?

C8 RESPONSE: The literature search did not impose any restrictions on publication date, allowing for the inclusion of studies published at any time to comprehensively capture the use of clinic-based technologies for assessing physical function in breast cancer survivors. This approach ensured that both early and recent studies were captured, providing a complete overview of the evolution and application of these technologies in clinical practice.

Tables 1 and 2 – You might move these tables to supplementary files. They’re lengthy and not very effective.

C9 RESPONSE: Done. Table 1 moved to supplementary files and table 2 removed and its content added to the main text.

Inclusion and Exclusion – I recommend standardizing your terminology in favor of “physical function” to describe your outcomes of interest. The last half of this paragraph might better be moved to the Introduction to help explain the rationale for your review (outcomes of interest). Your description of the technologies isn’t really a description of the inclusion/exclusion criteria. Also, use of “crucial” to describe real-time feedback needs evidence to support it. You seem to be saying that patient assessment and rehabilitation can’t be done without out. Where is the evidence?

C10 RESPONSE: Physical function used in the entire of the paper, and the definition and details of advance clinical-based technologies moved to introduction part.

The last half of this paragraph might better be moved to the Introduction to help explain the rationale for your review (outcomes of interest).

Revised content: Accordingly, this review aims to map and characterize the use of such advanced, clinically based technologies in assessing key physical functions, including balance, muscle strength, and range of motion (ROM), among individuals surviving breast cancer. For the purposes of this review, “advanced” is defined as instruments that provide automated, objective outputs beyond unaided observation or analogue readouts. “Clinic-based” denotes systems that can be operated in clinical rooms or clinically configured spaces by routine clinical staff with minimal specialist engineering support, for monitoring and assessing physical functions. These terms were selected based on their relevance within the ICF framework and their frequent association with impairment among breast cancer survivors [13,25,26]. By examining how these technologies are currently integrated into clinical practice, this review seeks to identify gaps in the existing literature and highlight areas where further research is needed.

Screening and Selection – Please be more precise. What do you mean by “most” of these studies? How many met the criteria? I’m trying to follow your search description in relation to Figure 1 and I can’t find any notation of where the change occurred in relation to the articles identified. Please be more explicit and provide a precise description of the search process that indicates where the change took place and how it modified your search results.

C11 RESPONSE: Revised Screening and Selection Process

An initial search strategy was developed in collaboration with the academic librarian to capture studies using any technology to assess physical function in adults with breast cancer. During the title and abstract screening process, it became evident that this comprehensive approach would yield several hundred eligible studies, predominantly due to the extensive literature on accelerometers and pedometers, as well as the frequent application of laboratory-based marker-based motion-capture systems. To preserve methodological rigor and avoid redundancy with existing systematic reviews and meta-analyses focused on accelerometer and pedometer studies, while ensuring a clinically actionable synthesis, an additional consultation with the librarian was conducted to refine the eligibility criteria prior to initiating full-text review and data extraction. The inclusion criteria were subsequently refined to prioritize advanced, clinically deployable technologies for point-of-care assessment of three core domains of physical function: balance, muscle strength, and range of motion. As a result, articles that used traditional technologies like dynamometers or goniometers as they do not meet the established definition of advanced measurement technology for the purposes of this review, non-clinic-based accelerometers and pedometers, and some lab-based motion capture systems requiring reflective markers, multi-camera stereophotogrammetry, calibration routines, and dedicated laboratory infrastructure were excluded unless authors explicitly described clinical deployment or adaptation for real-world clinical settings. The study selection process was conducted in several stages following established scoping review methodology. Initially, two reviewers (M.A. and A.W.) independently screened titles and abstracts of all retrieved references against the eligibility criteria. Discrepancies between the two reviewers at this stage were flagged within Covidence for subsequent resolution. Studies deemed potentially eligible by either reviewer advanced to full-text screening, which was similarly conducted independently by the same two reviewers (M.A. and A.W.). Following both screening phases, conflicts were systematically resolved through a structured consensus process. For any study where the two primary reviewers disagreed on inclusion or exclusion, the full text and relevant eligibility criteria were reviewed collaboratively by all three reviewers (M.A., A.W., and M.F.P.) in a consensus meeting. During these discussions, each reviewer presented their rationale, and discrepancies were resolved through deliberation until unanimous agreement was reached. If consensus could not be achieved through discussion, the third reviewer (M.F.P.) served as the final arbiter. All final inclusion decisions and data extraction were completed through this consensus process, with regular meetings held among the three reviewers to ensure consistency in interpretation of eligibility criteria and data extraction procedures throughout the review.

-I’m trying to follow your search description in relation to Figure 1 and I can’t find any notation of where the change occurred in relation to the articles identified.

Annotated box is added in the PRISMA diagram “Eligibility criteria refined after initial screening”

and how it modified your search results. The answer of this part is mentioned in the Screening and selection process in the main text.

4. Results

Study identification – Having full-text availability online isn’t part of your Results. I think it’s part of your Methods – maybe the inclusion/exclusion criteria.

C12 RESPONSE: Done.

Table 3 – This table isn’t readable. It needs to be fully reformatted to ensure readability. I recommend using landscape orientation.

C13 RESPONSE: Based on the instruction of the journal unfortunately I can not put the landscape table but I revised the table to be more readable.

Characteristics – You need to be more precise in reporting your findings. Be specific as to the number of studies you found for each point you make. Try to avoid terms like “many” or “some” that aren’t quantifiable. For example, you say that experimental methods were “most prevalent” but you identify only 5 out of 17 experimental studies. That’s only 30%. How is 30% considered most prevalent? The majority (12 out of 17) were apparently observational studies, so wouldn’t observational studies be the most prevalent? And when you identify a specific study, cite it so readers can easily match it to your reference list and Table 3. When describing participants, please include gender or sex. Were all participants females or were males also included? Last, what do you mean by “common exclusion criteria” and how were they used to guarantee participant safety and the integrity of the data?

C14 RESPONSE: Thank you for your constructive feedback. We have revised this section to remove vague adjectives.

The review included five experimental studies: three randomized controlled trials [28,33,38], one pretest– post-test experimental study [29], and one pilot intervention; [44] and twelve observational studies: four cross-sectional studies [32,34,35,40], three case-control studies [36,42,43], two prospective observational studies [30,31], two longitudinal cohort studies [37,39], and one descriptive study [41].

Among the 15 studies that reported participant sex, all included only female participants except for one study, which included a single male participant [37]. Two studies did not report any information about participant sex.

Nine of the 17 included studies (53%) explicitly reported participants' cancer stage; all nine enrolled individuals with stage 0–III disease [28,32–34,37,41–44]. . Five studies involved participants undergoing or having recently completed chemotherapy [29,32–34,43], and one study specifically enrolled women receiving hormone therapy [28]. One study focused on surgical reconstruction (LD flap) [30]. Two studies included post-mastectomy survivors compared to healthy controls [31,34], and three enrolled mixed-treatment cohorts [29,32,42]. In terms of sample sizes, these varied considerably across the studies, ranging from as few as 20 participants to as many as 100.

People with stage IV cancer and cognitive impairments were generally excluded. Additional exclusion criteria, such as uncontrolled cardiovascular or musculoskeletal conditions, severe neuropathy, or inability to provide informed consent, were applied to ensure participant safety and maintain the validity of assessment results.

Measurement Tools and Key Findings – This section reports your main findings. Cite each study as you identify and discuss it. What is the point of Figure 2? It just seems to report percentages of study outcome measures? Do you really need it? Couldn’t you just as effectively report in text that 90% of studies reported ROM as a measurement?

C15 RESPONSE: Cite each study as you identify and discuss it:

This scoping review found that most studies assessed physical function using a variety of outcome measures, ROM was reported in 8 of the 17 included studies (47.1%) [29,35,36,38–41,43]. Balance outcomes were reported in 5 studies (29.4%) [31,32,34,37,42], and muscle strength outcomes were reported in 4 studies (23.5%) [28,30,33,44]. Of these, three studies employed motion capture technologies [35,40,41], Kinect-based systems to assess upper extremity kinematics and ROM in individuals surviving breast cancer. Inclinometers were used in five studies to further quantify shoulder movements [29,36,38,39,43]. Balance assessments were another key focus, with multiple studies using advanced tools to evaluate postural stability and fall risk. Two studies employed force plates to measure postural sway in both static and dynamic conditions [32,37], while the SOT was used in another two studies to assess balance under varying sensory inputs [34,42].

Additionally, strength assessments were conducted using different models of the Biodex System (2, 3, 4, and 4 Pro) to evaluate isometric and isokinetic strength in muscle groups including the shoulder, hip, knee, and wrist [28,30,33,44].

Figure 2 is deleted, and the content is reported in the text. This scoping review found that most studies assessed physical function using a variety of outcome measures, Range of motion (ROM) was reported in 8 of the 17 included studies (47.1%) [26,32,33,35–38,40]. Balance outcomes were reported in 5 studies (29.4%) [28,29,31,34,39], and muscle strength outcomes were reported in 4 studies (23.5%) [25,27,30,41].

5. Discussion

Avoid terms like “vital” and “crucial” unless you have evidence that something can’t be done without whatever it is you’re saying is vital or crucial or whatever term you’re using.

C16 RESPONSE: the statement has been deleted.

Balance – Your description of the results of your review (for example predominant use of force plates, etc.) should be included in your Results. Discuss the implications of the findings here (why use of force plates is clinically important), but report the actual results in the previous Results section.

C17 RESPONSE: Studies that used advanced, clinic-based balance technologies, instrumented assessments targeted different aspects of postural control and consistently yielded information that simple, single-condition tests may miss. SOT protocols revealed impairments under proprioceptive and vestibular challenging conditions, despite near-normal performance on easier conditions, indicating that breast cancer survivorship related balance deficits can be context-dependent rather than global [34,42]. This pattern supports the clinical value of multi-condition batteries that probe sensory reweighting, rather than relying solely on single-task screens. Force plates quantify quiet-stance stability by deriving center of pressure (CoP) signals and summarizing them with standard sway metrics, typically medial–lateral (ML) and anterior–posterior (AP) excursion (range or root mean square (RMS)), total path length, mean sway velocity, and planar sway area (often a 95% confidence ellipse). These metrics capture both the magnitude (e.g., excursion, area) and temporal dynamics (e.g., velocity) of postural control. Notably, key ML CoP metrics including mean velocity, mean amplitude, and RMS displacement, have been linked to higher prospective fall risk [45]. In the included longitudinal study of taxane-based chemotherapy, Monfort et al. [37] used a laboratory force plate to track changes in CoP behavior during treatment; cumulative taxane exposure was associated with deteriorations in balance control, reflected by increased sway and concurrent gait alterations (shorter step length, slower walking speed). Complementing this, Wechsler et al. [32] showed that cancer-related fatigue independently predicted poorer postural stability on force-plate measures, consistent with compensatory stabilization strategies and greater sway under static and dynamic conditions. Together, these findings suggest that when clinically relevant stressors are present (neurotoxic chemotherapy, fatigue), force-plate CoP metrics are sensitive to subtle balance changes.

I have some concern regarding your use of References #53 and #54 that come from a commercial website (kinetisense.com). The content and postings on commercial websites aren’t peer reviewed and so they’re not a credible source, especially for comparisons of precision.

C18 RESPONSE: We agree that commercial websites are not appropriate sources for peer-reviewed scientific manuscripts, particularly when discussing measurement precision and validity. We have removed References #53 and #54 and eliminated the related content from the manuscript.

Muscle Strength – The sentence in paragraph 1 beginning “These effects...” doesn’t make sense. Is there a word or words missing? The last two sentences of this paragraph might be more effective in the Introduction as a rationale for muscle strength as an outcome of interest.

C19 RESPONSE: The paragraph is revised. Muscle strength was quantified with isokinetic dynamometry in controlled clinical or laboratory settings [28,30,33,44]. Systems such as the Biodex (models 2–4/4 Pro) are widely regarded as criterion instruments for dynamic muscle assessment because they impose precise, preset angular velocities and capture full torque–angle–velocity relationships together with work and power outputs. These multidimensional profiles can reveal deficits in force production and velocity-dependent behavior (e.g., concentric vs. eccentric weakness) that are not apparent from single-value manual grades, and their test–retest reliability in musculoskeletal [47,48]. The trade-off is practical, true isokinetic testing requires dedicated equipment, space, regular calibration, and staff skilled in positioning, stabilization, familiarization, and protocol standardization, all of which limit routine deployment outside well-resourced centers. Given these constraints, many rehabilitation services rely on portable HHDs for strength assessment. Professional guidance from the APTA Oncology EDGE Task Force recognizes HHDs as an appropriate option for cancer populations, provided that clinicians use standardized patient and tester positioning, consistent lever arms, and repeated trials to improve reliability [17].

The last two sentences of this paragraph might be more effective in the Introduction as a rationale for muscle strength as an outcome of interest. Done and removed to the introduction: Women undergoing chemotherapy may experience up to a 25% loss in strength and joint dysfunction [9].

Paragraph 2 – The identification and description of studies that used the Biodex system should be included in your Results. Please cite the de Lima et al. (2020) study to which you refer. Also, reference #66 doesn’t match the first author you identified (Drouin). Do you have any evidence that use of isokinetic machines offer unparalleled precision? I didn’t find any results regarding precision. Reference #71 doesn’t support your statement about chemotherapy reducing ROM and strength. The study didn’t include participants who had chemotherapy.

C20 RESPONSE: Also, reference #66 doesn’t match the first author you identified (Drouin). The problem was related to the Zotero citation.

Revised content For ROM measurement the review documents a technology continuum from operator-dependent digital inclinometers widely feasible but with persistent measurement limitations to automated MMC systems offering three-dimensional, bias-reduced assessment with emerging evidence of clinical utility. The successful application of MMC for upper extremity ROM assessment in breast cancer survivors, demonstrated across three studies in diverse settings including oncology clinics and outpatient centers, suggests these technologies have overcome initial feasibility barriers and may be ready for broader clinical implementation [35,40,41]. The strong correlation between Kinect-derived reachable workspace metrics and QuickDASH, a validated and widely used tool for assessing upper extremity disability in this population [46], while the system's ability to detect side-to-side differences in functionally relevant movement zones (overhead reaching) demonstrates practical utility for monitoring recovery [35]. Nonetheless, substantial heterogeneity in measurement protocols, encompassing whether motion is assessed actively versus passively, the specific planes or tasks evaluated, and the choice of absolute versus side-to-side metrics, complicates cross-study synthesis and limits the development of coherent, evidence-based clinical guidelines. Taken together, within the bounds of the included studies, MMC appears to have promising potential for assessing upper-extremity function in breast cancer survivors.

-Reference #71 doesn’t support your statement about chemotherapy reducing ROM and strength. The study didn’t include participants who had chemotherapy. The content is revised without using that reference:
